# ATG9A and ARFIP2 cooperate to control PI4P levels for lysosomal repair

**DOI:** 10.1016/j.devcel.2025.05.007

**Published:** 2025-05-27

**Authors:** Stefano De Tito, Eugenia Almacellas, Daniel Dai Yu, Emily Millard, Wenxin Zhang, Cecilia de Heus, Christophe Queval, Javier H. Hervás, Enrica Pellegrino, Ioanna Panagi, Ditte Fogde, Teresa L. M. Thurston, Judith Klumperman, Maximiliano Gutierrez, Sharon A. Tooze

**Affiliations:** 1 Molecular Cell Biology of Autophagy Laboratory, The Francis Crick Institute, London, UK; 2 Section Cell Biology, Center for Molecular Medicine, University Medical Center Utrecht, Utrecht University, Utrecht, the Netherlands; 3 High Throughput Screening, The Francis Crick Institute, London, UK; 4 Host-Pathogen Interactions in Tuberculosis Laboratory, The Francis Crick Institute, London, UK; 5 Department of Infectious Disease, Centre for Bacterial Resistance Biology, Imperial College London, London, UK; 6 Cell Death and Metabolism, Danish Cancer Institute, Copenhagen, Denmark; 7 Sir William Dunn School of Pathology, University of Oxford, Oxford, UK

## Abstract

Lysosome damage activates multiple pathways to prevent lysosome-dependent cell death, including a repair mechanism involving endoplasmic reticulum (ER)-lysosome membrane contact sites, phosphatidylinositol 4-kinase-2a (PI4K2A), phosphatidylinositol-4 phosphate (PI4P), and oxysterol-binding protein-like proteins (OSBPLs) lipid transfer proteins. PI4K2A localizes to the *trans*-Golgi network and endosomes, yet how it is delivered to damaged lysosomes remains unknown. During acute sterile damage and damage caused by intracellular bacteria, we show that ATG9A-containing vesicles perform a critical role in delivering PI4K2A to damaged lysosomes. ADP ribosylation factor interacting protein 2 (ARFIP2), a component of ATG9A vesicles, binds and sequesters PI4P on lysosomes, balancing OSBPL-dependent lipid transfer and promoting the retrieval of ATG9A vesicles through the recruitment of the adaptor protein complex-3 (AP-3). Our results identify a role for mobilized ATG9A vesicles and ARFIP2 in lysosome homeostasis after damage and bacterial infection.

## Graphical abstract

**Graphical abstract:**
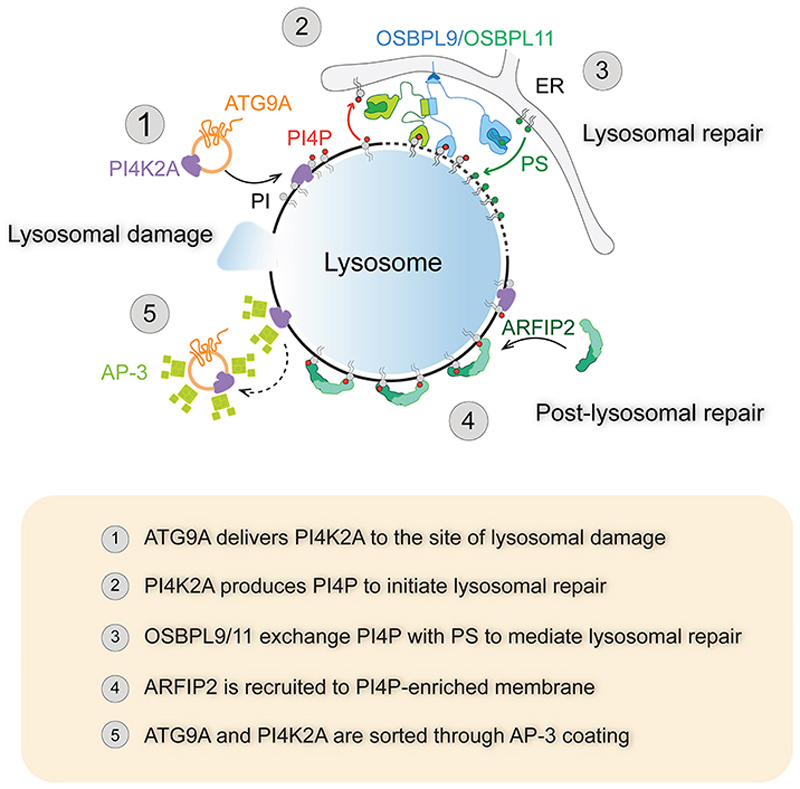

## Introduction

Lysosomal membrane permeabilization (LMP) is a process by which lysosomal membranes become leaky due to membrane damage. LMP has profound implications for neurodegenerative diseases, infection, and cancer.^1–4^ To counteract LMP and restore membrane integrity, cells have evolved several repair mechanisms. Under physiological conditions, LMP can be constrained by the endosomal sorting complex required for transport (ESCRT) machinery.^5^ Additionally, the phosphoinositide-initiated membrane tethering and lipid transport (PITT) pathway has been described to support lysosomal repair.^6^ Ruptured lysosomes can recruit Annexin 1 and 2^7^ in a Ca^2+^-dependent and ESCRT-independent fashion to prevent lysosomal leakage. Likewise, stress granules can accumulate at the damage site and stabilize the lysosomal membrane utilizing ESCRT-dependent or independent pathways.^8,9^ Inadequate lysosomal repair leads to lysophagy, a selective form of autophagy triggered by the ubiquitination of lysosomal proteins.^10^ In extreme cases, when damage is extensive, lysosome-dependent cell death is activated by the release of cathepsins into the cytosol.^11^ How these pathways are regulated and coordinated to promote survival is still an outstanding open question.

In the PITT pathway, phosphatidylinositol 4-kinase-2a (PI4K2A) is recruited to lysosomes to produce phosphatidylinositol-4 phosphate (PI4P), which recruits PI4P-binding proteins (OSBPL9, OSBPL10, OSBPL11, and OSBP) to establish endoplasmic reticulum (ER)-lysosome membrane contact sites that mediate lipid transfer for lysosomal repair.^6,12^ ATG2, a lipid transfer protein required for autophagy, is also recruited to lysosomes upon damage and contributes to lysosomal repair.^6^ Notably, ATG8s, in particular LC3A, can be lipidated on damaged lysosomes together with ATG2, independently of Uncoordinated-51-like kinase (ULK1/2) and WIPI2.^13^ Although PI4K2A initiates the PITT pathway via PI4P production, how it is delivered and regulated on damaged lysosomes remains largely unknown.

ATG9A, a lipid scramblase primarily studied for its function in autophagy, cycles between the *trans*-Golgi network (TGN) and endosomal compartments under normal conditions.^14^ During starvation, ATG9A traffics in vesicles from the TGN to ER-proximal sites where ATG13 and components of the ULK1 complex are concomitantly recruited.^15^ These ATG9A vesicles contain PI4KB, PI4K2A, and ADP ribosylation factor interacting protein 2 (ARFIP2),^16^ among other proteins.^17,18^ ARFIP2 is a Bin/Amphiphysin/Rvs (BAR)-domain-containing protein able to sense and induce membrane curvature.^19,20^ Through its BAR domain, ARFIP2 interacts with the small GTPases ADP-ribosylation factors (ARFs), ADP-ribosylation factor-like proten 1 (ARL1), and Ras-related C3 botulinum toxin substrate 1 (RAC1).^21–24^ Moreover, ARFIP2 contains an amphipathic helix (AH), which specifically binds PI4P in *in vitro* membrane models, and together with the BAR domain, ensures the correct TGN localization of ARFIP2.^25^ Further, ARFIP2 regulates the secretion of matrix metalloproteases 2 and 7 in complex with ARF1, ARL1, and serine/threonine-protein kinase D2 (PKD2),^26^ and positively modulates autophagy.^16^

ATG9A trafficking between different membrane compartments is coordinated by adaptor protein (AP) complexes.^27^ ATG9A appears to be the main cargo of Golgi-derived AP-4-coated vesicles, which are targeted to a vesicular compartment closely associated with autophagosomes.^28,29^ AP-2 binding supports ATG9A trafficking through the endosomal compartment, contributing to phagophore formation.^30^ Beyond autophagy, ATG9A has alternative functions in other membrane compartments, such as plasma membrane repair,^31^ lipid droplet homeostasis,^32^ and Golgi function.^33^ Interestingly, together with its localization at the TGN and early endosomes, ATG9A has been described more recently to transiently interact with lysosomes.^34,35^ Intriguingly, and relevant to the PITT pathway, ATG9A vesicles harbor PI4K2A,^16^ and ATG9A interacts with ATG2 and modulates its lipid transfer activity *in vitro*,^36,37^ prompting us to explore the potential involvement of ATG9A and the ATG9A-vesicle protein ARFIP2 in lysosomal repair mechanisms. Here, we uncover a function of ATG9A vesicles as PI4K2A carriers to mediate lysosomal repair in response to acute sterile damage and damage caused by intracellular bacteria. In this context, we reveal a dual role for ARFIP2 in lysosomal damage response. On one hand, it regulates lysosomal repair by binding PI4P on the lysosomes, restricting its availability for the oxysterol-binding protein-like protein (OSBPL)-mediated lipid transfer between ER-lysosome contact sites needed for lysosomal repair. On the other hand, ARFIP2 orchestrates the sorting of ATG9A vesicles and PI4K2A away from the lysosomes by recruiting adaptor protein complex-3 (AP-3), which we identified as the adaptor complex needed for ATG9A retrieval.

## Results

### ATG9A is recruited to lysosomes upon lysosomal damage

**Figure 1:**
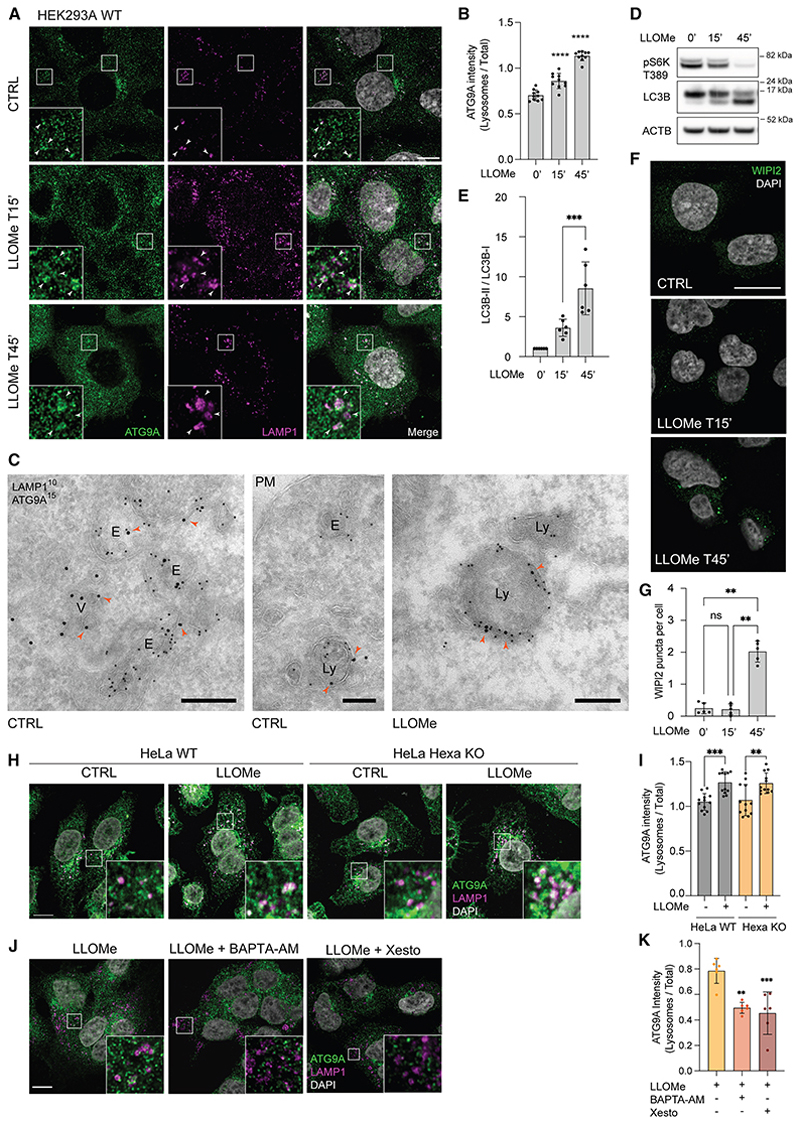
ATG9A is recruited to lysosomes upon lysosomal damage (A) Immunofluorescence of HEK293A WT (CTRL) treated with 1 mM LLOMe for the indicated times. Scale bar: 10 μm. (B) Quantification of (A). *n* = 5 independent experiments. (C) Double immunogold labeling of LAMP1 (10 nm gold) and ATG9A (15 nm gold, arrows) in control and LLOMe-treated cells. ATG9A is predominantly found in small vesicles (V) and occasionally on the limiting membrane or in the lumen of endosomes (E) and lysosomes (Ly). Scale bars: 200 nm. (D) Western blot of HEK293A WT cells treated with 1 mM LLOMe for the indicated times. (E) Quantification of LC3-II/LC3-I intensity ratio. *n* = 6 independent experiments. (F) Immunofluorescence of HEK293A WT treated with 1 mM LLOMe for the indicated times. Scale bar: 10 μm. (G) Quantification of (F). *n* = 5 independent experiments. (H) Immunofluorescence of HeLa WT and Hexa KO cells treated with 1 mM LLOMe for 15 min. Scale bar: 10 μm. (I) Quantification of (H). *n* = 3 independent experiments. (J) Immunofluorescence of HEK293A treated or not with BAPTA-AM (50 μM) or xestospongin C (10 μM) for 30 min before treatment with LLOMe for 15 min. Scale bar: 10 μm. (K) Quantification of (J). *n* = 5 independent experiments. Bar graph data are shown as mean ± SD. Statistical significance is noted as: **p < 0.005, ***p < 0.001, ****p < 0.0001. See also Figure S1.

To investigate ATG9A trafficking upon lysosomal damage, we followed ATG9A localization upon acute lysosomal damage induced by the lysosomotropic agent L-leucyl-L-leucine methyl ester (LLOMe). Interestingly, ATG9A disperses from the perinuclear region and shows an increase on the lysosome associated membrane protein 1 (LAMP1)-positive membranes upon LLOMe treatment (Figures 1A, 1B, S1A, and S1B). To visualize ATG9A at the ultrastructural level, we performed immunogold labeling followed by electron microscopy. Double labeling of ATG9A and endogenous LAMP1 demonstrates that while the vast majority of ATG9A resides in small vesicles, ATG9A can be detected on the limiting membrane of endosomes and lysosomes positive for LAMP1 both in control and upon LLOMe treatment (Figure 1C). Multiple ATG9A vesicles (V) can be found clustered around endolysosomes (E and Ly), some of which are in close proximity, suggesting tethering and possibly fusion or budding events (Figures 1C and S1C). As autophagy induction triggers ATG9A exit from the TGN and dispersal into vesicles, we tested whether acute lysosomal damage is triggering autophagy and therefore mobilization of ATG9A. Lysosome damage has been shown to inactivate the mammalian target of rapamycin complex 1 (mTORC1),^8^ thus stimulating macroautophagy. To assess mTORC1 activity, we measured the phosphorylation of S6K (T389), a downstream target. Importantly, while mTORC1 activity was unaffected after 15 min of LLOMe, it was inhibited at later time points (Figures 1D and S1D). As ATG13 translocates within seconds to ATG9A-positive structures,^15^ it is unlikely that ULK1 activation dependent on mTORC1-inactivation is involved in ATG9A trafficking to lysosomes upon damage. LC3B lipidation, a hallmark of autophagy, was detectable by 15 min after LLOMe treatment, and its levels drastically increased after 45 min in parallel to the reduction in mTORC1 activity (Figures 1D and 1E). In addition, we used WIPI2 as a bona fide early autophagy marker. After 15 min of LLOMe treatment, the number of WIPI2 spots did not increase, further ruling out autophagy initiation as the trigger for ATG9A mobilization (Figures 1F and 1G). Upon lysosomal damage, LC3B lipidation can be induced by the conjugation of ATG8s to single membranes (CASMs), a pathway that is independent of canonical autophagy,^13^ and LC3B lipidation on lysosomes can trigger the association of ATG2. As ATG9A interacts with ATG2,^36^ we tested whether ATG8s were mediating ATG9A recruitment to lysosomes upon damage. In HeLa Hexa knockout (KO) cells, which lack all ATG8s,^38^ ATG9A recruitment to lysosomes upon acute damage still occurs (Figures 1H and 1I), suggesting that its recruitment is independent of CASM. Importantly, LLOMe treatment did not significantly affect ATG9A levels (Figures S1E and S1F). Finally, we explored whether Ca^2+^ efflux triggered by lysosomal damage could promote the recruitment of ATG9A vesicles to the lysosomal compartment. During LLOMe treatment, the use of the membrane-permeable fast Ca^2+^ chelator 1,2-bis(2-aminophenoxy)ethane-N,N,N′,N′-tetraacetic acid tetrakis(acetoxymethyl ester) (BAPTA-AM) reduced ATG9A colocalization with lysosomes (Figures 1J and 1K). Furthermore, inhibition of ER Ca^2+^ release upon LLOMe treatment by xestospongin C induced a similar phenotype (Figures 1J and 1K).^39^ Conversely, activating the transient receptor potential cation channel mucolipin 1 (TRPML1), a lysosomal Ca^2+^ channel, with its agonists MK6-83 and ML-SA1 enhanced ATG9A colocalization with lysosomes, similar to LLOMe treatment (Figures S1G and S1H). These findings suggest that Ca^2+^ plays a pivotal role in driving ATG9A vesicles to the lysosome upon lysosomal damage. In parallel to a reduction in ATG9A vesicle recruitment to the lysosome upon damage, both BAPTA-AM and xestospongin C impaired lysosomal repair, leading to increased accumulation of LGALS3 spots (Figures S1I and S1J). Given the relevance of lysosomal integrity in neurons,^40^ we tested ATG9A lysosomal colocalization in a more physiological model using SH-SY5Y neuroblastoma cells differentiated into neuron-like cells prior to LLOMe treatment (Figures S1K and S1L). ATG9A vesicles were mobilized to lysosomes upon damage in distinct ring-like structures decorating LAMP1-positive membranes (Figure S1M). Together, these data support that lysosomal damage induces a Ca^2+^-dependent ATG9A vesicle mobilization to the damaged lysosome.

### ARFIP2 loss increases lysosomal ATG9A, promoting lysosomal repair

**Figure 2:**
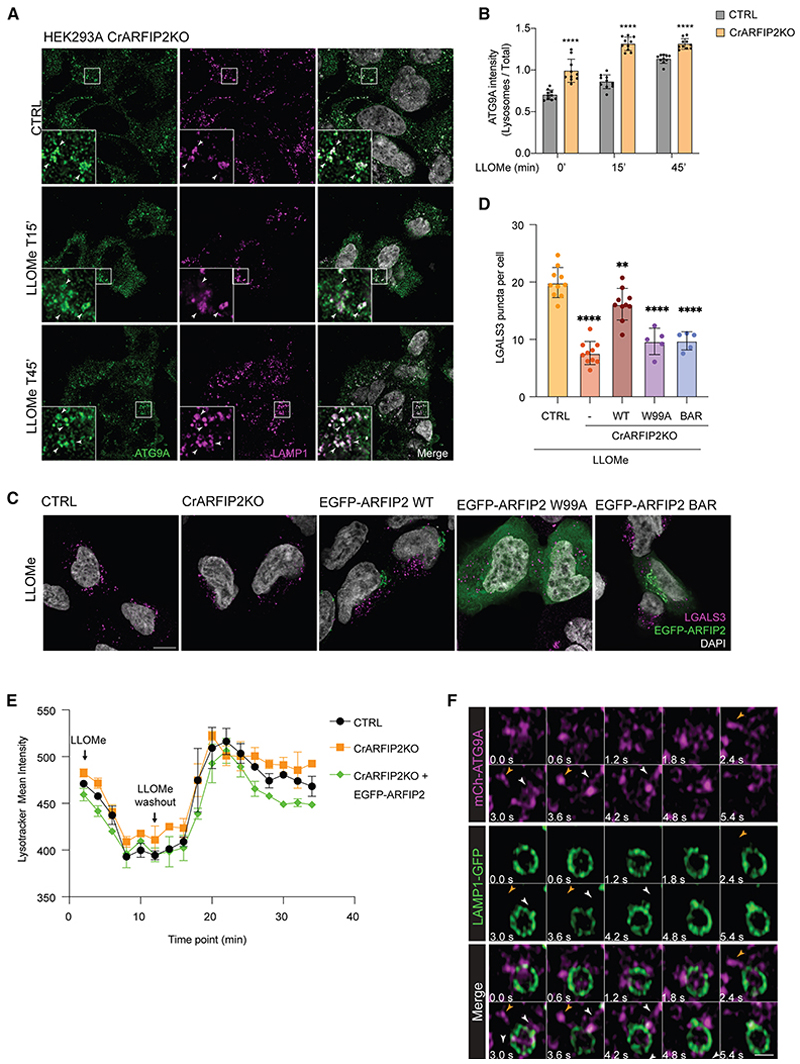
ARFIP2 controls ATG9A lysosomal localization for lysosomal damage (A) Immunofluorescence of CrARFIP2KO cells treated with 1 mM LLOMe for the indicated times. Scale bar: 10 μm. (B) Quantification of (A). *n* = 5 independent experiments. (C) Immunofluorescence of control (CTRL) or CrARFIP2KO cells transfected with EGFP-ARFIP2, EGFP-ARFIP2^W99A^ mutant, or EGFP-BAR and treated with LLOMe 1 mM for 15 min. Scale bar: 10 μm. (D) Quantification of (C). *n* = 5 independent experiments. (E) LysoTracker recovery assay of CTRL, CrARFIP2KO, and CrARFIP2KO cells transfected with EGFP-ARFIP2. *n* = 1 experiment as an average of 2 replicates and 6 fields per replicate. (F) Frames of live imaging experiments, at the indicated time points, of CrARFIP2KO cells transfected with LAMP1-GFP and mCherry-3xFLAG-ATG9A. Arrowheads indicate tubulation events. Scale bar: 1 μm. Bar graph data and line plot are shown as mean ± SD. Statistical significance is noted as: **p < 0.005, ****p< 0.0001 See also Figure S2 and Video S2.

Dispersal of ATG9A from its TGN localization was previously found upon loss of ARFIP2.^16^ Thus, we tested whether ARFIP2 might regulate ATG9A localization to the lysosomes. Interestingly, an enrichment of ATG9A on the lysosomal compartment was detected in HEK293A CrARFIP2KO compared with control (CTRL) cells, which was further enhanced upon LLOMe treatment (Figures 2A and 2B). Note, acute LLOMe treatment did not alter ARFIP2 total protein levels (Figures S2A and S2B). We then investigated whether this increased lysosomal recruitment of ATG9A in CrARFIP2KO cells might impact lysosomal damage or repair. Galectin-3 (LGALS3) accumulation was used to detect damage to lysosomal membranes. Upon LLOMe treatment, CrARFIP2KO cells exhibited fewer LGALS3 spots than CTRL cells, and this effect was reversed by overexpressing EGFP-ARFIP2 wild type (WT) (Figures 2C, 2D, S2C, and S2D). As ARFIP2 interacts with PI4P via its AH, we tested whether the effects observed upon damage were dependent on the association of ARFIP2 to PI4P. Expression of ARFIP2 W99A AH mutant, which is unable to bind to PI4P,^25^ failed to increase LGALS3 spots, indicating that ARFIP2 binding to PI4P is required for its function in lysosomal damage (Figures 2C and 2D). To further characterize which ARFIP2 region is required for the lysosomal damage response, we overexpressed only its BAR domain (109–341). Importantly, overexpression of the EGFP-BAR domain alone was insufficient to restore the phenotype, indicating that both PI4P binding and the ARFIP2 N-terminal region are critical for this function (Figures 2C and 2D). Finally, to determine whether PI4P itself is important for lysosomal repair upon ARFIP2 loss, we blocked PI4P availability by overexpressing the PI4P probe P4M-SidM from *Legionella pneumophila* prior to LLOMe treatment. PI4P blockade by P4M increased LGALS3 spots upon acute damage, supporting the key role of PI4P for lysosomal repair both in CTRL and CrARFIP2KO cells (Figures S2E and S2F). To distinguish lysosomal damage from repair, we analyzed LysoTracker fluorescence recovery after LLOMe treatment.^41^ LysoTracker fluorescence requires lysosomal acidification; therefore, decreased fluorescence reflects lysosomal damage, which can be restored upon repair. We found that fluorescence recovery upon LLOMe washout occurred faster and to a greater extent in CrARFIP2KO cells than in CTRL or EGFP-ARFIP2-rescued cells (Figures 2E and S2G). Furthermore, we tested whether the ESCRT protein CHMP4B was also differentially recruited to damaged lysosomes in CrARIP2KO cells upon LLOMe. Our results showed increased CHMP4B recruitment to damaged lysosomes, further supporting an increased repair upon loss of ARFIP2 (Figures S2H and S2I). As ARFIP2 loss increases ATG9A colocalization with lysosomes, and to further investigate the interaction between ATG9A vesicles and the lysosomes, we performed live imaging experiments in CrARFIP2KO cells overexpressing mCherry-3xFLAG-ATG9A and GFP-LAMP1. While the majority of ATG9A vesicles transiently interact with the lysosomes, a pool of ATG9A was detected enriched on specific micro-domains on larger lysosomes (Figure S2J; Video S1) and was able to interact with the LAMP1-positive compartments and undergo tubulation (Figure 2F; Video S2).

### AP-3 is an ARFIP2 interactor that regulates ATG9A trafficking through the endolysosomal compartment

**Figure 3:**
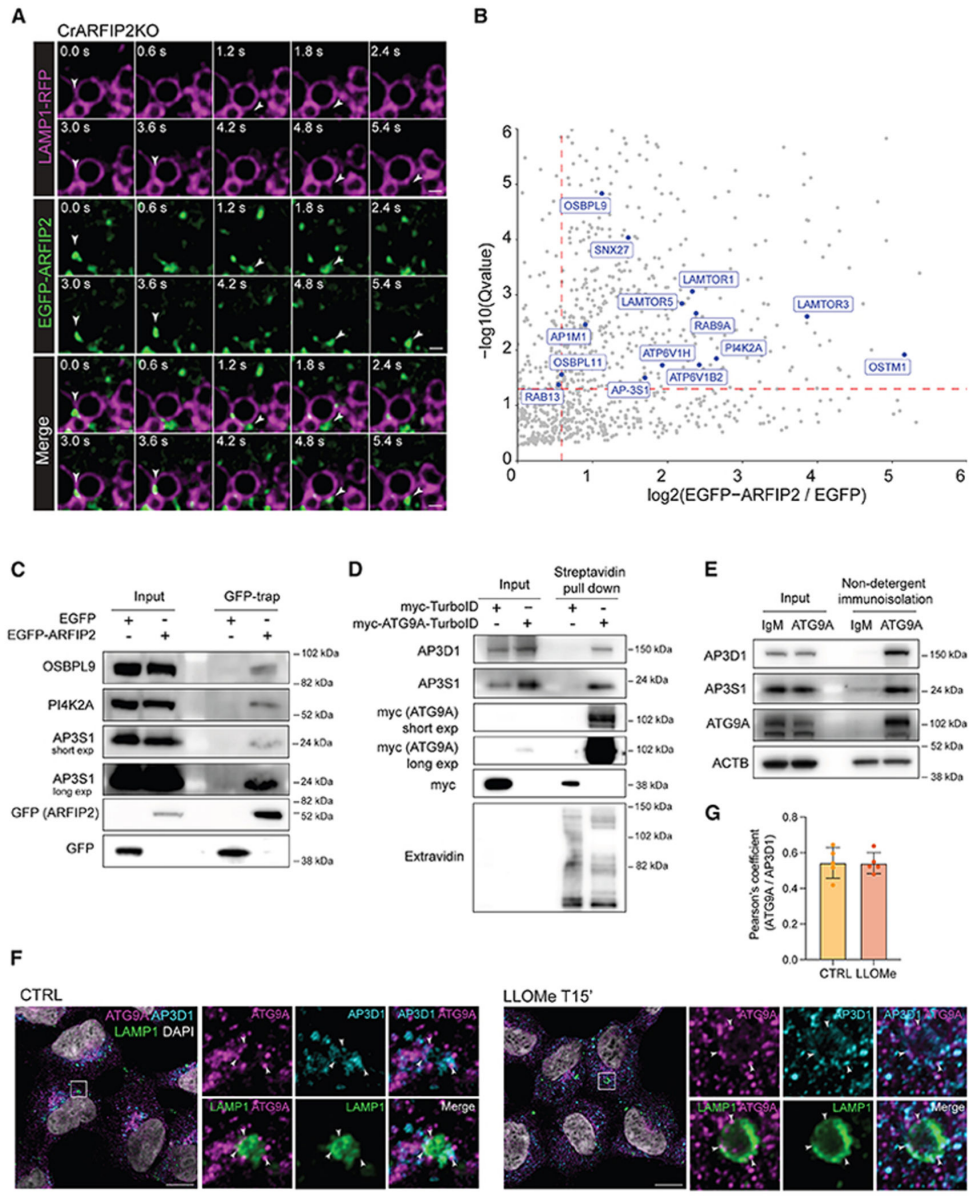
ARFIP2 interacts with lysosomal proteins and AP-3 (A) Live imaging of CrARFIP2KO transiently transfected with EGFP-ARFIP2 (green) and LAMP1-RFP (magenta). Arrowheads indicate colocalization events. Scale bar: 1 μm. (B) Label-free mass spectrometry of GFP-trap performed in CrARFIP2KO cells stably expressing EGFP or EGFP-ARFIP2. Lysosomal proteins (data from Cell Map^42^), proteins of the PITT pathway, and AP proteins are labeled in the volcano plot. (C) Validation was performed by GFP-trap. (D) Western blot of CrATG9AKO cells stably expressing myc-TurboID or myc-ATG9A-TurboID subjected to streptavidin pull-down. (E) Western blot of ATG9A-positive membranes immunoisolation. (F) Immunofluorescence of HEK293A cells. Scale bar: 10 μm. (G) Quantification of Pearson correlation between ATG9A and AP3-D1. Bar graph data are shown as mean ± SD. See also Figure S3 and Video S3.

We further explored the mechanism underlying ATG9A increase on lysosomes upon ARFIP2 loss. Although ARFIP2 has been described as a Golgi-resident protein,^25^ live-cell imaging experiments showed that EGFP-ARFIP2 can localize to lysosomes positive for RFP-LAMP1 (Figure 3A; Video S3). Mass spectrometry experiments of immunoprecipitated EGFP-ARFIP2 stably expressed in CrARFIP2KO cells identified several lysosomal proteins and proteins belonging to the PITT pathway, as well as the adaptor complex (AP) AP-3 (Figures 3B and 3C; Table S1). AP-3 is involved in the sorting of transmembrane proteins to/from the endolysosomal compartment.^43–45^ Despite various AP complexes (AP-1, AP-2, AP-4) previously being implicated in regulating ATG9A trafficking,^28,29,46,47^ a potential role for AP-3 was not known. An ATG9A-proximal proteome generated from CrATG9AKO cells stably expressing myc-ATG9A-TurboID mass spectrometry analysis and western blot analysis revealed that two subunits of the AP complex AP-3 (AP3B1 and AP3S1) are ATG9A interactors (Figure 3D; Table S2). Immunoisolation experiments of endogenous ATG9A-positive membranes further support this finding (Figure 3E). We confirmed that myc-ATG9A-TurboID colocalizes with and behaves like endogenous ATG9A, redistributing into peripheral cytosolic vesicles upon nutrient starvation (Figures S3A and S3B). Additionally, immunofluorescence analysis demonstrated similar colocalization of AP-3 and ATG9A both in basal conditions and upon lysosomal damage (Figures 3F and 3G). Co-staining with the lysosomal marker LAMP1 showed that AP-3 and ATG9A partially overlap with the lysosomal compartment (Figure 3F, insets). Since the depletion of a single subunit of an AP complex disrupts the formation and stability of the tetrameric complex,^48^ we silenced the AP-3 sigma subunit (AP3S1) in HEK293A cells and found in fed cells this led to ATG9A dispersal (Figures S3C–S3E). Importantly, AP3S1 depletion did not significantly alter ARFIP2 localization on the lysosomes (Figures S3F and S3G). Conversely, ARFIP2 loss did not affect AP-3 localization (Figures S3H and S3I).

**Figure 4:**
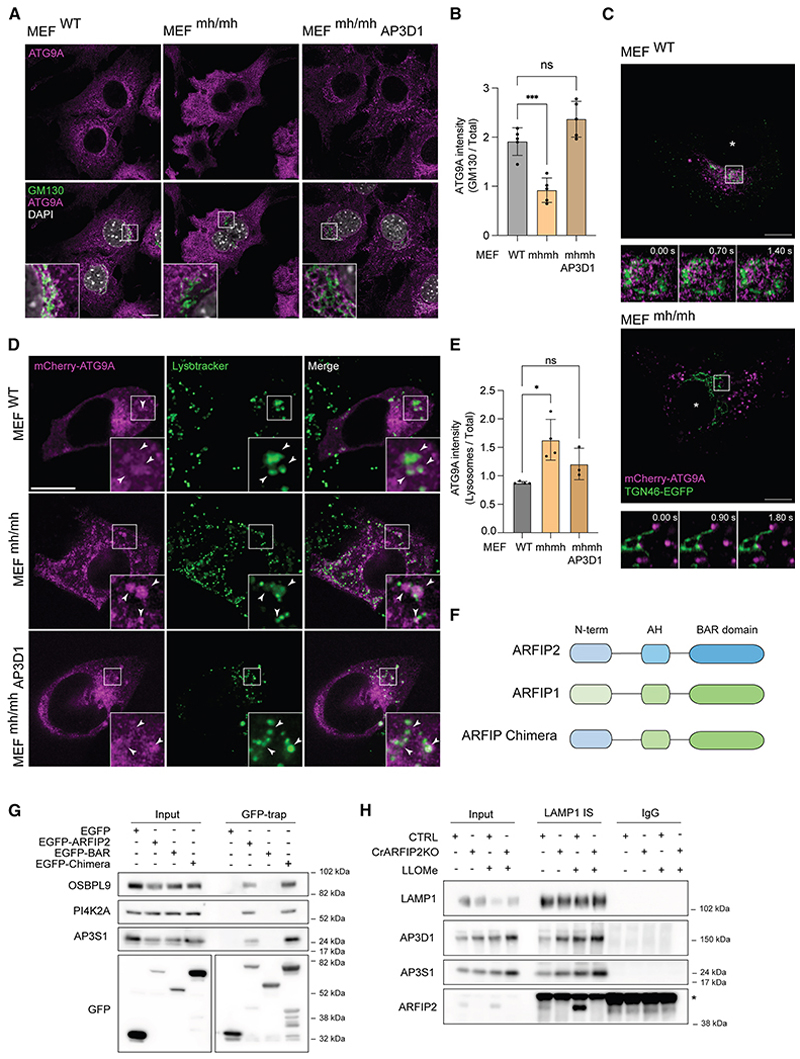
AP-3 controls ATG9A trafficking throughout the endolysosomal compartment (A) Immunofluorescence of MEF^WT^, MEF^mh/mh^, and MEF^mh/mh^ stably expressing AP-3D1. Scale bar: 10 μm. (B) Quantification of ATG9A in the Golgi from (A). *n* = 5 independent experiments. (C) Live imaging of MEF^WT^ and MEF^mh/mh^ transiently expressing TGN46-EGFP and mCherry-3xFLAG-ATG9A. Representative panels of *n* = 10 cells. (D) Live imaging of MEF^WT^, MEF^mh/mh^, and MEF^mh/mh^ stably expressing AP-3D1, transiently transfected with mCherry-3xFLAG-ATG9A and loaded with LysoTracker blue DND-26 (green) for 1 h. Scale bar: 10 μm. (E) Quantification of (D). *n* = 4 fields were analyzed per condition. (F) Schematics of ARFIP2 WT, ARFIP1 WT, and ARFIP chimera. (G) GFP-trap of CrARFIP2KO cells stably expressing EGFP, EGFP-ARFIP2, EGFP-AH-BAR (BAR), and EGFP-ARFIP chimera. (H) Western blot of LAMP1-positive membranes immunoisolation in CTRL and CrARFIP2KO cells treated or not with LLOMe 1 mM for 15 min. Bar graph data are shown as mean ± SD. Statistical significance is noted as: *p < 0.05, ***p < 0.001. See also Figure S4 and Video S4.

To validate the missorting of ATG9A upon loss of AP-3, we took advantage of fibroblasts from the *mocha* mouse model (MEF^mh/mh^), which are AP-3 deficient due to the loss of AP3D1^49^ (Figure S4A). Genetic depletion of AP-3 has been described as the underlying genetic cause of Hermansky-Pudlak syndrome type 10 (HPS10), which is characterized by severe phenotypes such as inner ear degeneration, pigmentation dysfunctions, and neurological deficits.^49^ ATG9A localization was significantly altered in MEF^mh/mh^ mouse embryonic fibroblasts compared with WT (MEF^WT^), with an accumulation of ATG9A in cytosolic vesicular structures in MEF^mh/mh^ cells (Figures 4A and 4B). Importantly, overexpression of the AP3D1 subunit in these cells rescued the expression of the full AP-3 complex as well as ATG9A localization (Figures 4A, 4B, S4A, and S4B). ATG9A dispersal in MEF^mh/mh^ cells was corroborated by differential centrifugation experiments (Figures S4C–S4E). Comparison of MEF^mh/mh^ with MEF^WT^ revealed an enrichment of ATG9A in the lightest membrane fraction, in parallel with a decrease in heavier ones (Figures S4D and S4E). Interestingly, the levels of the lower band of ATG9A, corresponding to the ER-specific form,^50^ were unchanged in the MEF^mh/mh^, suggesting that the AP-3 complex is not involved in ATG9A trafficking between the ER and the Golgi apparatus (Figure S4D). Live imaging experiments, using mCherry-3xFLAG-ATG9A and GFP-TGN46 to label the Golgi compartment in MEF^WT^ or MEF^mh/mh^ cells, provided dynamic insights into ATG9A constitutive trafficking. In contrast to MEF^WT^, ATG9A displayed a more dispersed localization, barely colocalizing with the TGN in MEF^mh/mh^ cells, suggesting an impairment in the trafficking and/or recycling of ATG9A-positive vesicles (Figure 4C; Video S4).

In addition to the Golgi complex, ATG9A localizes to the endocytic-recycling compartment (ERC) and endosomes^17,30,51–55^ and partially with the lysosomal compartment,^34,35^ supporting the notion that ATG9A constitutively traffics throughout the endolysosomal system. Given the role of the AP-3 complex, we investigated whether ATG9A relocalized to the endolysosomal compartment in MEF^mh/mh^ cells. MEF^WT^ and MEF^mh/mh^ cells, expressing mCherry-3xFLAG-ATG9A, were incubated with fluorescent LysoTracker for 1 h to visualize the endolysosomal compartment. In live-cell imaging, the amount of ATG9A colocalizing with the endolysosomal compartment was significantly higher in MEF^mh/mh^ compared with MEF^WT^, suggesting a defect in ATG9A sorting (Figures 4D and 4E). Importantly, cells re-expressing AP3D1 rescued this phenotype, supporting a role for AP-3 in ATG9A retrieval from the endolysosomal system (Figures 4D and 4E).

As ARFIP2 interacts with the AP-3 complex and regulates ATG9A localization, we investigated which domain of ARFIP2 is involved in the interaction with AP3S1. GFP-trap experiments showed that ARFIP2 full-length, but not its BAR (109–341) domain (GFP-BAR), interacts with AP3S1 (Figures 4F and 4G). In contrast to the ARFIP2 BAR domain, the N-terminal ARFIP2-ΔBAR (1–108) domain does not retain its Golgi localization upon overexpression, hindering its functional characterization (Figure S4F). As the BAR domains of ARFIP1 and 2 are highly conserved,^25^ and ARFIP1 is not involved in ATG9A vesicle trafficking,^16^ we exploited this to test the role of the N-terminal domain of ARFIP2. We engineered an ARFIP chimera by fusing the N-terminal portion of ARFIP2 (1–92) with the BAR domain of ARFIP1 (93–341) (Figures 4F and S4F). Similar to ARFIP2, ARFIP chimera localizes mainly in the Golgi and can interact with AP3S1, while the ARFIP2 AH-BAR domain did not, demonstrating that the N-terminal portion of ARFIP2 is responsible for its interaction with AP3S1 (Figure 4G). Surprisingly, two other ARFIP2 interactors, OSBPL9 and PI4K2A, involved in the PITT pathway also specifically interacted with the N-terminal domain (Figure 4G). These results align with the requirement of the N-terminal domain of ARFIP2 for the lysosomal damage response (Figures 2C and 2D). Finally, by immunoisolating LAMP1-positive membranes, we confirmed the presence of both ARFIP2 and AP-3 on the lysosomes, both of which are enriched upon lysosomal damage (Figure 4H).

As ARFIP2 interacts with AP-3 through its N-terminal portion, these data support a model whereby ARFIP2, bound to PI4P on membranes via the AH-BAR, interacts with AP-3 through its N-terminal domain to retrieve ATG9A from lysosomes. The increased ARFIP2 levels on LAMP1 immunoisolated membranes upon LLOMe treatment support its crucial role during the process of lysosomal damage/repair.

### ATG9A coordinates PI4K2A delivery to the lysosomes for lysosomal repair

**Figure 5:**
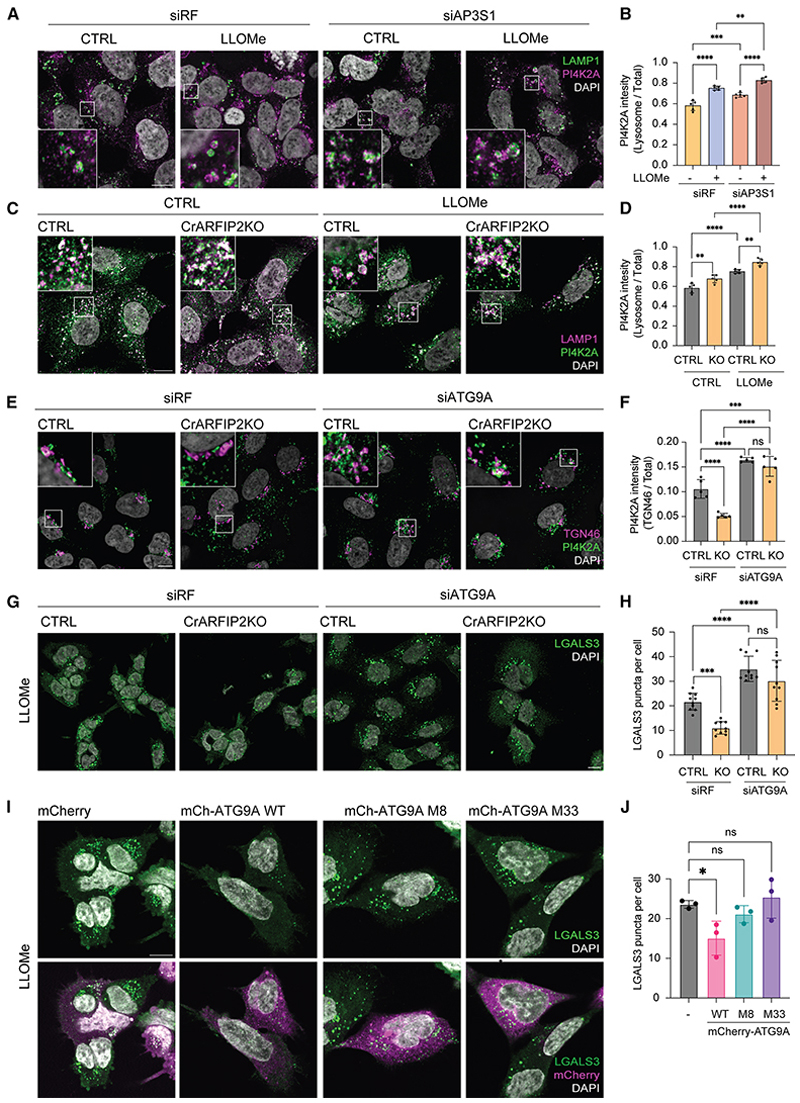
ATG9A and ARFIP2 control PI4K2A trafficking and subsequent lysosomal repair (A) Immunofluorescence of Risc-free (siRF) or siAP-3S1#1 treated with 1 mM LLOMe for 15 min. Scale bar: 10 μm. (B) Quantification of (A). *n* = 5 independent experiments. (C) Immunofluorescence of CTRL and CrARFIP2KO cells treated with 1 mM LLOMe for 15 min. Scale bar: 10 μm. (D) Quantification of (C). *n* = 5 independent experiments. (E) Immunofluorescence of CTRL or CrARFIP2KO cells transfected with siRF or siRNA ATG9A. Scale bar: 10 μm. (F) Quantification of (E). *n* = 5 independent experiments. (G) Immunofluorescence of siRF or siATG9A in CTRL or CrARFIP2KO cells treated with 1 mM LLOMe for 15 min. Scale bar: 10 μm. (H) Quantification of (G). *n* = 5 independent experiments. (I) Immunofluorescence of CrATG9AKO transfected with mCherry, mCherry-ATG9A WT, or the scramblase mutants M8 and M33 and treated with LLOMe 1 mM for 15 min. (J) Quantification of (I). *n* = 3 independent experiments. Bar graph data are shown as mean ± SD. Statistical significance is noted as: *p < 0.05, **p < 0.005, ***p < 0.001, ****p < 0.0001. See also Figure S5.

AP-3 and PI4K2A interact, and both the dileucine motif and kinase activity of PI4K2A are required for AP-3 and PI4K2A localization to the lysosomal compartment.^56–58^ Indeed, cells lacking AP-3 show an accumulation of PI4P in the lysosomes that is rescued by overexpression of the AP-3 complex (Figures S5A and S5B). Moreover, PI4K2A accumulates in the lysosomal compartment upon loss of AP-3 (Figures 5A and 5B). Thus, we investigated whether loss of ARFIP2 affects the intracellular distribution of PI4K2A. Similar to ATG9A, PI4K2A was more dispersed and accumulated in the lysosomal compartment in CrARFIP2KO cells (Figures 5C and 5D). PI4K2A dispersal was also supported by differential centrifugation experiments that showed enrichment of both PI4K2A and ATG9A in the lightest membrane fraction in CrARFIP2KO cells compared with CTRL cells (Figures S5C–S5E). Moreover, PI4K2A accumulation was validated by immunoisolation of LAMP1-positive membranes upon LLOMe treatment (Figure S5F). Concomitantly, the colocalization between ATG9A and PI4K2A increased upon LLOMe treatment (Figures S5G and S5H). While ARFIP2 loss increases the presence of both PI4K2A and ATG9A in the lysosomal compartment, loss of PI4K2A by small interfering RNA (siRNA) in HEK293A did not alter ATG9A lysosomal recruitment upon LLOMe, indicating that PI4K2A is downstream of ATG9A and ARFIP2 (Figures S5I–S5K). Considering that PI4K2A associates with ATG9A membranes,^16^ we tested if PI4K2A mislocalization in CrARFIP2KO cells requires ATG9A. ATG9A siRNA knockdown (KD) induced an accumulation of PI4K2A in the peri-Golgi area both in CrARFIP2KO and CTRL cells, supporting the function of ATG9A-positive vesicles as carriers for PI4K2A delivery toward the endolysosomal compartment (Figures 5E, 5F, and S5L). To determine whether lysosomal repair induced by loss of ARFIP2 depends on ATG9A, we silenced ATG9A in either CTRL or CrARFIP2KO cells and analyzed LGALS3 spots upon LLOMe treatment. Loss of ATG9A increased the number of LGALS3 spots in CTRL and CrARFIP2KO, supporting a role for ATG9A in regulating lysosomal damage (Figures 5G and 5H). Accordingly, the LysoTracker recovery assay showed that ATG9A loss impaired lysosomal repair even to a similar extent as overexpression of EGFP-ARFIP2 WT in CrARFIP2KO cells (Figure S5M). ATG9A has been described to function as a lipid scramblase, which is important for autophagy and phagophore expansion. Therefore, we tested whether the ability of ATG9A to scramble lipids is important for its function in lysosomal repair. To test this hypothesis, we generated two previously described mutants that are defective for the scramblase activity (M8 and M33).^59^ We overexpressed these mutants alongside the WT ATG9A and treated cells with LLOMe. Our data showed that, while the WT ATG9A could rescue the increased damage observed in CrATG9AKO cells, both M8 and M33 scramblase mutants were unable to do so (Figures 5I and 5J).

In summary, our findings indicate that ARFIP2 plays a crucial role in regulating the localization of PI4K2A and ATG9A at the lysosomal compartment, which contributes to lysosomal repair. Furthermore, ATG9A functions as a critical upstream regulator of PI4K2A trafficking, with its lipid scramblase activity being indispensable for lysosomal repair. These results underscore the coordinated interplay between ARFIP2, ATG9A, and PI4K2A in maintaining lysosomal integrity and repairing damage.

### ARFIP2 acts as a key regulator of PI4P production and lipid transfer for lysosomal repair

**Figure 6:**
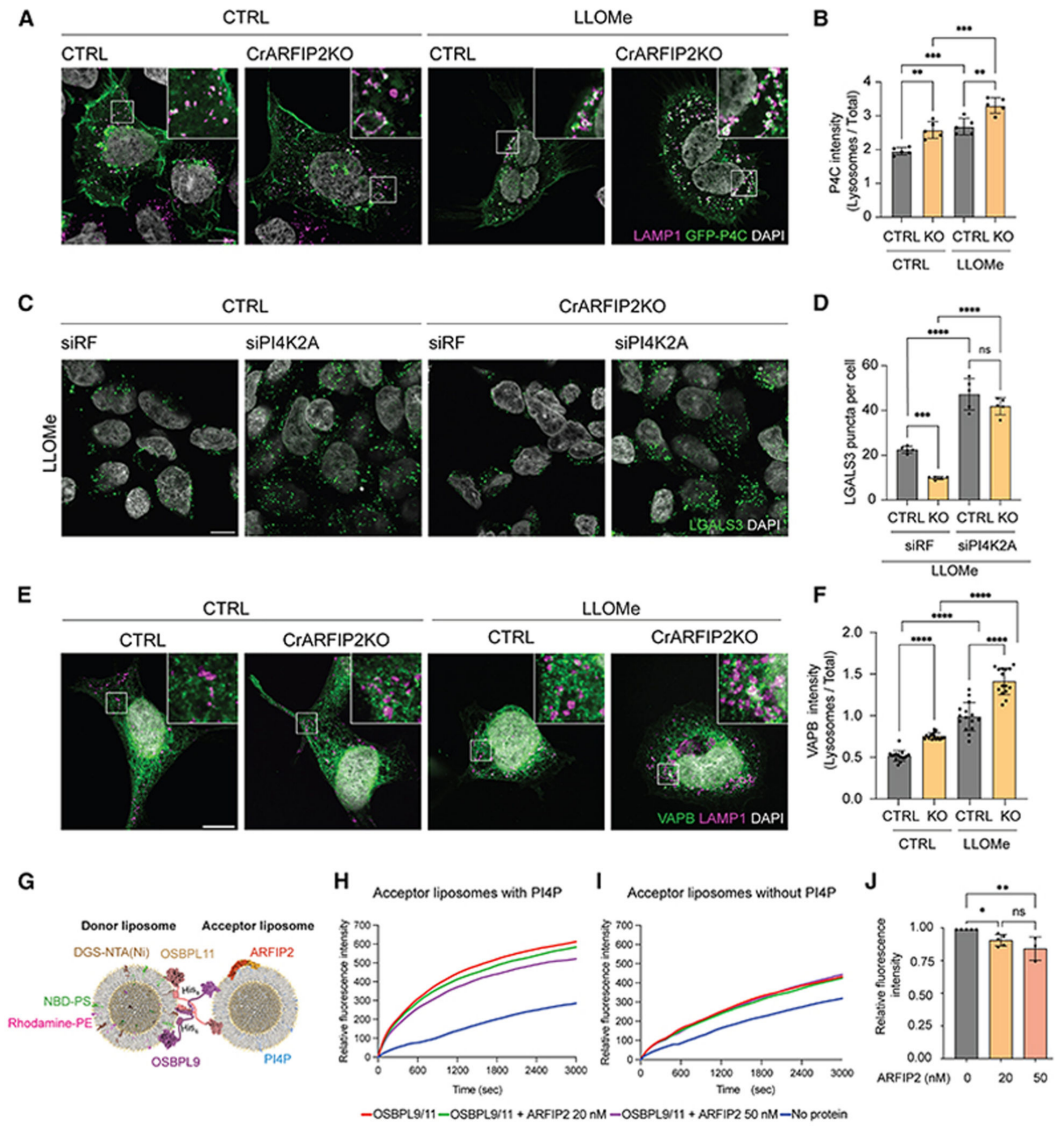
Enhanced lysosomal repair in CrARFIP2KO correlates with elevated PI4P lysosomal production (A) Immunofluorescence of CTRL and CrARFIP2KO cells expressing GFP-P4C-SidC and treated with 1 mM LLOMe for 15 min. Scale bar: 10 μm. (B) Quantification of (A). *n* = 5 independent experiments. (C) Immunofluorescence of siRF or siPI4K2A in CTRL or CrARFIP2KO cells treated with 1 mM LLOMe for 15 min. Scale bar: 10 μm. (D) Quantification of LGALS3 spots from (C). *n* = 5 independent experiments. (E) Immunofluorescence of CTRL and CrARFIP2KO cells treated with 1 mM LLOMe for 15 min. Images were acquired using a SoRa super-resolution microscope followed by deconvolution. Scale bar: 10 μm. (F) Quantification of VAPB intensity on the lysosomal compartment from (E). *n* = 3 independent experiments. (G) Schematic model of the *in vitro* lipid transport assay. The structure models for ORP9/11 heterodimer and ARFIP2 homodimer were obtained using AlphaFold/ AlphaFold multimer modeling. (H and I) PS transfer activity of ORP9/11 heterodimer in the presence or absence of ARFIP2 using acceptor liposomes with (H) or without PI4P (I). (J) PS transfer activity of ORP9/11 heterodimer in the presence or absence of ARFIP2 with PI4P-containing acceptor liposomes. *n* > 3 independent experiments. Bar graph data are shown as mean ± SD. Statistical significance is noted as: *p < 0.05, **p < 0.005, ***p < 0.001, ****p < 0.0001. See also Figure S6 and Video S5.

PI4K2A is the main PI4P-producing enzyme at the lysosomes and has a prominent role in lysosomal repair.^6,12^ Indeed, the lysosomal accumulation of PI4K2A in CrARFIP2KO cells reflected an increased level of PI4P, as detected by the specific probe GFP-P4C-SidC^60^ (Figures 6A and 6B; Video S5), likely contributing to a more efficient repair of damaged lysosomes. Importantly, PI4P accumulation depends on ATG9A, as loss of ATG9A reduced the amount of PI4P at the lysosomal compartment concomitantly to a defect in PI4K2A trafficking (Figures S6A and S6B). Manipulation of PI4P levels by depletion of PI4K2A or chemical inhibition using NC03 promoted lysosomal damage detected by LGALS3 spots both in WT and CrARFIP2KO cells (Figures 6C, 6D, S6C, and S6D). Likewise, overexpression of SACM1L, a phosphatase that metabolizes PI4P, counteracted the effects of ARFIP2 depletion and prevented lysosomal repair (Figures S6E and S6F), while depletion of SACM1L reduced lysosomal damage (Figures S6G–S6I). Therefore, our findings suggest that upon damage, ATG9A vesicles relocate to lysosomes to deliver PI4K2A, which drives PI4P production and mediates lysosomal repair upon damage.

Lysosomal repair requires ER-lysosome contact sites where lipid transfer can occur through OSBPLs and OSBP, which bind PI4P. Specifically, PI4P is exchanged with phosphatidylserine (PS) or cholesterol by OSBPL9-10-11 heterodimers and OSBP, respectively.^61^ Both OSBPL9 and OSBP contain an FFAT (two phenylalanines in an acidic tract) domain that anchors them to the ER via interaction with the vesicle-associated membrane protein-associated proteins A and B (VAPA/B). Thus, we investigated whether the increased lysosomal PI4P production in CrARFIP2KO cells leads to elevated ER-lysosome contact sites. As expected, super-resolution microscopy revealed an increased clustering of the VAPB-positive compartment around the lysosomes following LLOMe treatment (Figures 6E and 6F). Interestingly, VAPB enrichment on lysosomes was even more pronounced in CrARFIP2KO cells compared with CTRL cells, with or without LLOMe (Figures 6E and 6F).

As the ARFIP2 AH-BAR domain specifically binds PI4P,^25^ and PI4P accessibility is important for efficient repair (Figures S2E and S2F), we hypothesized that ARFIP2 might bind the increased PI4P produced by PI4K2A, thus interfering with OSBPL-mediated lipid transfer. To test this, we used fluorescence resonance energy transfer (FRET)-based *in vitro* lipid transport assays to demonstrate that the OSBPL9/11 heterodimer transports PI4P (Figure 6G), and such transport is increased when the donor lipid compartment is enriched in PI4P (Figures 6H and 6I). Interestingly, the addition of ARFIP2 significantly reduces OSBPL9/11 lipid transport when the donor liposomes were enriched in PI4P, likely through direct protein-lipid interaction (Figure 6J).

Overall, our findings suggest that PI4K2A-mediated PI4P production controls the formation of ER-lysosome contact sites, facilitating lipid exchange and promoting efficient lysosomal repair. During this process, ARFIP2 emerged as a regulator of the PITT pathway by binding to PI4P, thereby reducing its availability for lipid exchange and fine-tuning OSBPLs-mediated lipid transfer.

### ARFIP2-dependent membrane repair pathway is required for *M. tuberculosis* and *Salmonella* restriction

**Figure 7:**
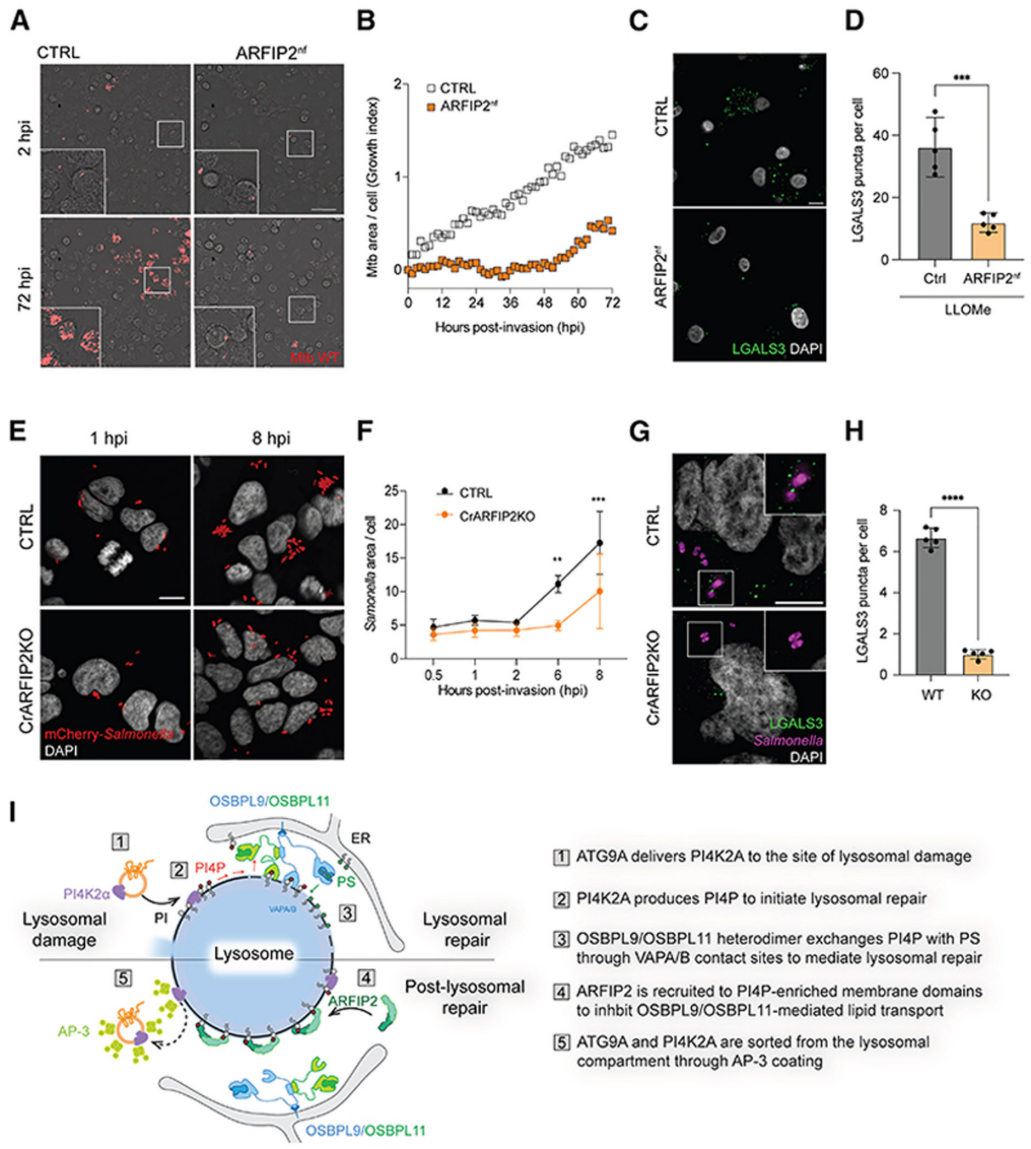
ARFIP2 loss restricts *Mycobacterium tuberculosis* and *Salmonella* infection (A) Snapshot of CTRL and ARFIP2^nf^ HMDM infected with *Mycobacterium tuberculosis* WT (red). Scale bar: 50 μm. (B) High-content quantitative analysis of live *Mycobacterium tuberculosis* WT replication in HMDM CTRL and ARFIP2^nf^. *n* = 2 independent experiments. (C) Immunofluorescence of CTRL and ARFIP2^nf^ HMDM cells treated with 1 mM LLOMe for 15 min. Scale bar: 10 μm. (D) Quantification of LGALS3 spots from (C). *n* = 5 independent experiments. (E) Representative images of mCherry-*Salmonella*-infected CTRL and CrARFIP2KO cells. Scale bar: 10 μm. (F) *Salmonella* growth was monitored as area *Salmonella* area per cell at the indicated time post infection. *n* = 5 independent experiments. (G) Immunofluorescence of mCherry-*Salmonella*-infected CTRL and CrARFIP2KO at 30 min post infection. Scale bar: 10 μm. (H) Quantification of LGALS3 spots from (G). *n* = 5 independent experiments. (I) Schematic model of ARFIP2-ATG9A-PI4K2A interplay in repairing damaged lysosomes. The five steps of the repair mechanism are described on the right. Bar graphs and line plots are shown as mean ± SD. Statistical significance is noted as: **p < 0.005, ***p < 0.001, ****p < 0.0001. See also Figure S7.

In the context of infection, some pathogens rupture membranes to infect the cytosol, where they can proliferate. To evaluate if the ARFIP2-dependent membrane repair pathway contributes to the restriction of intracellular pathogens, we used primary human monocyte-derived macrophages (HMDMs) infected with *Mycobacterium tuberculosis*. We confirmed that upon LLOMe treatment, ATG9A was recruited to the lysosomes in human macrophages (Figures S7A and S7B), revealing a conserved mechanism for ATG9A trafficking upon lysosomal damage. We nucleofected HMDM with Cas9 protein and single-guide RNA (sgRNA) targeting ARFIP2^62^ to obtain an ARFIP2 KO pool (ARFIP2^nf^) (Figures S7C–S7E). Strikingly, after *Mycobacterium tuberculosis* WT infection, there was enhanced bacterial restriction in ARFIP2^nf^ compared with CTRL cells (Figures 7A and 7B). In agreement with these results, a reduced number of LGALS3 spots was observed in ARFIP2^nf^ compared with CTRL macrophages upon LLOMe treatment (Figures 7C and 7D). These data indicate that loss of ARFIP2 contributes to the restriction of *Mycobacterium tuberculosis* infection.

We also investigated the effects of ARFIP2 loss in *Salmonella* infection. Despite both CTRL and CrARFIP2KO cells showing the same number of *Salmonella*-infected cells (Figure S7F), pathogen replication was more restricted in CrARFIP2KO cells compared with CTRL cells (Figures 7E and 7F). Further, CrARFIP2KO cells showed a reduced number of LGALS3 spots during *Salmonella* infection (Figures 7G and 7H), together with an increased association of ATG9A and PI4K2A with the bacteria (Figures S7G and S7H). Additionally, mCherry-*Salmonella* was engulfed in LAMP1-positive membranes in CrARFIP2KO cells (Figure S7I), confirming that the pathogen was restricted in the endolysosomal compartment upon ARFIP2 loss. Overall, these data support a role for ATG9A and PI4K2A recruitment to the site of pathogen infection to mediate membrane repair and restrict pathogen infection and a regulation of these events by ARFIP2.

## Discussion

Lipid metabolism has gained a central role in lysosomal repair, where damaged membranes require lipids for expansion and sealing. Lipid-metabolizing enzymes and membrane contact sites between lysosomes and other membrane compartments are now acknowledged as key factors that counteract LMP. Our work identifies ATG9A vesicles as membrane carriers delivering PI4K2A to damaged lysosomes, facilitating PI4P production, and activating the PITT pathway. Loss of ATG9A causes retention of PI4K2A in the Golgi, preventing its function at the lysosomes and increasing lysosomal damage. Structure-guided molecular simulations predicted that ATG9A induces membrane bending,^63^ supporting a plausible involvement in segregation of membrane domains prior to vesicle formation. Moreover, specific lipids, such as sphingomyelin, have been shown to prevent ATG9A traffic from the Golgi.^64^ This suggests that ATG9A may mediate PI4K2A trafficking by modifying the lipid environment, enabling its recruitment to specific Golgi subdomains prior to vesicle formation. Previous studies have shown that ATG9A regulates the trafficking of another PI-metabolizing enzyme, PI4KB,^16^ which can also localize at lysosomes.^65^ However, PI4P produced by PI4KB has been shown to be dispensable for lysosomal repair but relevant for lysosomal reformation coupled with ARF1 GTPase function.^65^

Our data ruled out autophagy initiation or ATG8s lipidation by CASM as triggers for ATG9A trafficking upon LMP. Recent studies have shown that Ca^2+^ efflux and transients regulate autophagy initiation.^66,67^ Consistent with this, our findings show that chelating Ca^2+^ with BAPTA-AM negatively impacts ATG9A recruitment, while activating the lysosomal Ca^2+^ channel TRPML1 enhances ATG9A localization to lysosomal membranes. However, how highly localized Ca^2+^ release either from the damaged lysosome or the ER would trigger ATG9A trafficking from the Golgi compartment is an outstanding question that remains to be addressed.

Our findings suggest transient interactions occur between ATG9A vesicles and the lysosomal compartment. High-resolution imaging and immunogold labeling confirmed that ATG9A primarily localizes to small vesicles, occasionally detectable in clusters^53,68^ and endolysosomes. We also detected ATG9A vesicles in close proximity to, and on, the limiting membrane of LAMP1-positive late endosomes and lysosomes. However, it remains uncertain whether ATG9A vesicles fuse with lysosomes or undergo a kiss-and-run interaction with the membrane, as shown for synaptic vesicles.^69^ Proteomics data from our laboratory^16^ and others revealed a diverse pool of soluble NSF-attachment protein receptors (SNAREs) on ATG9A vesicles, which could potentially be involved in the fusion of ATG9A vesicles with the lysosomes. Nonetheless, definitive proof demonstrating integration of ATG9A into the lysosome membrane, with or without damage, remains a challenge for future research. We have explored the importance of ATG9A scramblase activity for lysosomal repair. Importantly, we showed ATG9A scramblase activity, crucial for specific functions, such as autophagy,^59^ is required for lysosomal repair. However, studies of ATG9A function for plasma membrane repair found that the scramblase mutant was able to rescue the phenotype to the same extent as WT protein,^31^ suggesting a context-dependent scramblase requirement. *In vitro*, scramblase mutants show a 50% reduction in their scramblase activity, raising the possibility that specific functions of ATG9A are either independent of the lipid scrambling or only require partial scrambling efficiency.

Lysosome-ER contact sites and lipid transfer have been proposed to play critical roles in lysosomal repair. Upon lysosomal damage, galectins recruit ESCRT proteins to seal the damaged membranes by invagination of the damage and potential loss of membrane surface. ATG9A, via its scramblase activity, may facilitate the accessibility of lipids for OSBPL-mediated lipid transfer at ER-lysosome contact sites. Alternatively, ATG9A might bind ATG2, modulating its lipid transfer activity to fine-tune lipid flux needed for lysosomal repair. Both these mechanisms would cooperate with the ESCRT machinery to ensure lysosome membrane restoration upon damage.

Multiple complementary mechanisms operating during lysosomal damage and repair have been uncovered in recent years.^5–9,13^ However, the coordination and sequence of events involved remain unresolved. It is unclear how ESCRT-mediated repair, the PITT pathway, annexins, stress granule formation, and the sphingomyelin-dependent repair pathway cooperate in concert to mediate lysosomal repair or whether distinct signals can selectively trigger them. We describe here the interaction between ARFIP2 and lysosomes, which is increased upon acute LMP. Our results support a dual function for ARFIP2 in the control of lysosomal repair by (1) mediating PI4K2A retrieval from lysosomes through AP-3 and (2) modulating OSBPLs-mediated lipid transfer through PI4P binding. Consequently, ARFIP2 loss induces PI4K2A accumulation at the lysosomes, increasing PI4P production and enhancing lysosomal repair.

We propose a model where ARFIP2 senses high levels of PI4P via its AH-BAR domain after LMP, leading to the inhibition of PI4P lipid transfer mediated by OSBPL9-10-11 heterodimers and OSBP (Figure 7I). Concurrently, the ARFIP2 N-terminal domain, together with PI4K2A, promotes coat assembly on PI4P-enriched regions by interaction with AP-3. Furthermore, using different biochemical approaches, we validated AP-3 as an adaptor complex that decorates ATG9A vesicles, supporting our model. AP-1, AP-2, and AP-4 have also been described as regulators of ATG9A vesicle trafficking in other membrane compartments. ATG9A contains both a tyrosine and a dileucine motif that mediate these interactions. We could not detect a direct interaction between AP-3 and ATG9A, suggesting that other proteins, such as PI4K2A or ARFIP2, might bridge this association. In *C. elegans*, which lacks the AP-4 complex,^70^ ATG9A requires AP-3 for TGN exit into the synaptic vesicle cycle. However, whether mammalian AP-3 plays conserved roles with AP-4 in the post-Golgi trafficking of ATG9A remains unknown. Since, in all probability, different adaptor complexes cannot simultaneously coat the same vesicle, we suggest that each adaptor complex identifies a distinct pool of ATG9A devoted to compartment-specific functions.

Finally, we have translated the importance of this pathway to the physiological context of pathogen infection. During infection, host cell machineries target pathogens to phagosomes for endolysosomal degradation.^71^ However, several bacteria can damage the membrane of phagosomes and access the cytosol,^72^ making endolysosomal integrity crucial to restrict infection. ESCRT proteins are known to prevent lysosome rupture during infection.^73^ PI4P and OSBP also accumulate on *Mycobacterium tuberculosis*-containing vacuoles (MCVs), forming ER-contact sites that restore MCV integrity via ER-dependent repair.^74^ Our data demonstrate that loss of ARFIP2 enhances restriction of *Salmonella* infection, therefore impairing bacterial proliferation in HEK293A cells and *Mycobacterium tuberculosis* in human macrophages, revealing the broad significance of this repair mechanism across various cell types and pathogens. These data, along with the mobilization of ATG9A upon LMP induction in different models, warrant further investigation in other biological contexts where lysosomal damage and repair play a critical role, such as neurodegenerative diseases or cancer.

### Limitations of the study

Our data support the role for ARFIP2, a TGN resident protein, in the regulation of lysosomal repair by contributing to the retrieval and regulation of the endolysosomal repair machinery. ARFIP2 has been shown to localize on ATG9A-positive vesicles; however, it remains unclear whether ARFIP2 is relocalized to the lysosome upon damage via TGN trafficking, potentially through ATG9A vesicles.

Our results support a role for the AP-3 complex in the retrograde transport of both ATG9A and PI4K2A from the endolysosomes to the TGN. While a sorting motif for AP-3 recognition has been identified in PI4K2A, whether AP-3 directly interacts with ATG9A and the adaptor-related trafficking machinery, including the GTPase involved, remains unknown.

Finally, while the scramblase activity of ATG9A appears to be required for lysosomal repair, the specific lipid species translocated by ATG9A at the lysosome membrane remain to be explored. Furthermore, whether lipid transfer proteins, such as ATG2A or VPS13C, cooperate with ATG9A in lysosome repair remains to be addressed.

### Resource Availability

### Lead contact

Further information and requests for resources and reagents should be directed to and will be fulfilled by the lead contact, Sharon Tooze (Sharon.Tooze@crick.ac.uk).

### Materials availability

All materials generated in this study are available from the lead contact. This study did not generate new unique reagents.

## Star★Methods

Detailed methods are provided in the online version of this paper and include the following:

- KEY RESOURCES TABLE
- EXPERIMENTAL MODEL AND STUDY PARTICIPANT DETAILS
  - Cell culture
  - Preparation and culture of HMDMs
  - Mycobacterial strains and culture conditions
  - *Salmonella* strains and culture conditions
- METHOD DETAILS
  - Plasmids and siRNA transfection
  - Nucleofection of HMDM
  - Macrophage infection with *Mycobacterium tuberculosis*
  - Long-term live-cell imaging of *Mycobacterium tuberculosis* replication and HMDM
  - *Salmonella* infection
  - Proximity labelling using TurboID and streptavidin pull-down
  - GFP-trap immunoprecipitation
  - Cell lysis and Western Blot
  - Mass spectrometry LC-MS/MS and MS data processing and analysis
  - Immunofluorescence and confocal microscopy
  - Immunogold labelling and electron microscopy methods
  - Lysotracker uptake and recovery
  - Live imaging
  - Image analysis
  - Immunoisolation of ATG9A-positive membranes
  - Lysosome immunoprecipitation
  - Differential centrifugation for cell fractionation
  - Protein expression and purification
  - Liposome preparation and Lipid transfer assay
- QUANTIFICATION AND STATISTICAL ANALYSIS

## Star★Methods

### Key Resources Table

**Table:** 

REAGENT or RESOURCE	SOURCE	IDENTIFIER
Antibodies		
Rabbit Polyclonal ACTB	Abcam	Cat. #ab8227; RRID: AB_2305186
Mouse Monoclonal anti-delta SA4 (AP3D1)	Andrew Peden, School of Bioscience, University of Sheffield, Sheffield, United Kingdom	N/A
Rabbit polyclonal anti-delta hinge 1 (AP3D1)	Andrew Peden, School of Bioscience, University of Sheffield, Sheffield, United Kingdom	N/A
Rabbit Polyclonal AP3S1	Abcam	Cat. #ab113099; RRID: AB_10860326
Rabbit Polyclonal ARFIP2	Thermo Fisher Scientific	Cat. #40-2400; RRID: AB_2533458
Hamster Monoclonal ATG9A	CRUK	in-house 14F2
Rabbit Polyclonal ATG9A STO215	CRUK	in-house STO215
Rabbit Polyclonal ATG9A STO219	CRUK	in-house STO219
b-3 Tubulin	Thermo Fisher Scientific	Cat. #MA5-19187
Mouse Monoclonal EEA1	BD Biosciences	Cat. #610457; RRID: AB_397830
Mouse Monoclonal FLAG-M2	Sigma-Aldrich	Cat. #F3165; RRID: AB_259529
Mouse monoclonal GFP	CRUK (in-house)	Clone 3E10
Mouse Monoclonal GM130	BD Biosciences	Cat. #610823; RRID: AB_398142
Mouse Monoclonal CD107a (LAMP1)	BD Pharmigen	Cat. #555798; RRID: AB_396132
Rabbit monoclonal LAMP1 (D2D11)	Cell Signaling	Cat. #9091; RRID: AB_2687579
Rat Monoclonal LAMP1 (1D4B)	Abcam	Cat. #ab25245; RRID: AB_449893
Rabbit Polyclonal LC3B	Abcam	Cat. #ab48394; RRID: AB_881433
Rat Monoclonal LGALS3-Alexa 488 conjugated	Biolegend	Cat. #125410; RRID: AB_10730886
Rat Monoclonal LGALS3-Alexa 594 conjugated	Biolegend	Cat. #125412; RRID: AB_2565600
Rat Monoclonal LGALS3-Alexa 647 conjugated	Biolegend	Cat. #125408; RRID: AB_1186110
Mouse monoclonal Myc	CRUK (in-house)	Clone 9E10
Rabbit Recombinant Monoclonal OSBPL9 (ORP9)	Abcam	Cat. #ab249015
Mouse Monoclonal Phospho-p70 S6 Kinase (Thr389) (1A5)	Cell Signaling Technology	Cat. #9206; RRID: AB_2285392
Mouse Monoclonal PI4K2A	Santa Cruz	Cat. #sc-390026
Rabbit Polyclonal RAB5A	Abcam	Cat. #ab18211; RRID: AB_470264
Mouse Monoclonal Rab11	BD Biosciences	Cat. #610656; RRID: AB_397983
Rabbit Polyclonal Rab7	Cell Signaling	Cat. #2094; RRID: AB_2300652
Mouse Monoclonal Rab9	Abcam	Cat. #ab2810; RRID: AB_303323
Rabbit Polyclonal SACM1L	Proteintech	Cat #13033-1-AP; RRID: AB_2301284
Rabbit Monoclonal SAP97	Abcam	Cat. #ab134156
Rabbit Monoclonal SQSTM1	Cell Signaling Technology	Cat. #39749; RRID: AB_2799160
Rabbit Monoclonal SYP	Thermo Fisher Scientific	Cat. #MA5-14532; RRID: AB_10983675
Rabbit Polyclonal TGN46	Abcam	Cat. #ab50595; RRID: AB_2203289
Mouse Monoclonal TOM20	Abcam	Cat. #ab56783; RRID: AB_945896
Rabbit Polyclonal VAPB	ATLAS Antibodies	Cat. #HPA013144; RRID: AB_1858717
Mouse Monoclonal VCL	Sigma Aldrich	Cat. #V9264; RRID: AB_10603627
Mouse Monoclonal WIPI2	CRUK (in-house)	Clone A2A
Bacterial and virus strains		
*Mycobacterium tuberculosis* H37Rv *(Mycobacterium tuberculosis* WT)	Douglas Young, The Francis Crick Institute, UK	N/A
*Salmonella enterica* serovar Typhimurium, strain 14028s	Theresa L.M Thurston, Imperial College London	N/A
Chemicals, peptides, and recombinant proteins		
LLOMe	Cambridge Bio (BACHEM)	Cat. #4000725
Xestospongin C	Tocris/Bio-techne	Cat. #1280/10U
BAPTA-AM	Abcam	Cat. #ab120503
MK6-83	Tocris/Bio-techne	Cat. #5547/5547/10
ML-SA1	Tocris/Bio-techne	Cat. #4746/4746/10
LysoTracker Red DND-99	Life Tech/Fisher	Cat. #L7528
LysoTracker Green DND-26	Life Tech/Fisher	Cat. #L7526
LysoTracker Deep Red	Life Tech/Fisher	Cat. #L12492
Streptavidin Alexa 647 conjugated	Thermo Fisher Scientific	Cat. #S21374
Extravidin - Peroxidase	Merck	Cat. #E2886
GFP-trap	Chromotek	Cat. #gta-20
Binding Control Agarose	Chromotek	Cat. #bab-20
cOmplete EDTA free protease inhibitor tablets	Sigma-Aldrich	Cat. #11836170001
Phostop EASYpack	Roche	Cat. #04906837001
Hoechst	Sigma-Aldrich	Cat. #23491-45-4
anti-DYKDDDDK G1 affinity resin	GenScript	Cat. #L00432
3xFlag peptide	Produced in-house	N/A
FBS	Sigma-Aldrich	Cat. #F7524
Deposited data		
TurboID-ATG9A	PRIDE	PXD056417
GFP-ARFIP2 interactome	PRIDE	PXD056398
Experimental models: Cell lines		
Human: HEK293A	ATCC	Cat. #CRL-1573
Human: SH-SY5Y	Cell Services STP, The Francis Crick Institute	N/A
Human: Leucocyte cones	NHS Blood and Transplant service	NC24
Human: HeLa wt	ATCC	Cat. #CCL-2
Human: HeLa Hexa KO	Michael Lazarou, Walter and Eliza Hall Institute of Medical Research, Parkville, Victoria, Australia	
Mouse: MEF wt	Cell Services STP, The Francis Crick Institute	N/A
Mouse: *Mocha* fobroblast	ATCC	Cat. #CRL-2709
Oligonucleotides		
AIRFP2_sg1:ACACAATGCTG GTTTCATTG - AGG	Synthego	N/A
AIRFP2-sg4: GTCCTGACAC CATCACCTGC - TGG	Synthego	N/A
AIRFP2-sg3: CCCTGGAGAT GAGGTGGCTC – GGG	Synthego	N/A
AIRFP2-sg2: ATGGGGCATCA ACACCTATA – AGG	Synthego	N/A
siGenome RISC-Free Control	Horizon	Cat. #D-001220-01
siGenome ATG9A: UGACAGA ACUGGACAUCUA	Horizon	Cat. #D-014294-02
siGenome AP-3 #1: UGAAUG AGAUUGUUACACA	Horizon	Cat. #D-012569-01
siGenome AP-3 #2: UAGACAA GGUUCACAAUAU	Horizon	Cat. #D-012569-02
Custom siRNA PI4K2A: UGAAGC AGAACCUCUUCCUGAUU	Horizon	N/A
siGENOME Human SACM1L (22908) siRNA - SMARTpool	Horizon	Cat. #M-007280-00
Recombinant DNA		
pEGFP-C1	Clontech	N/A
EGFP-ARFIP2 WT	Zhiqiu Man and Kazuhisa Nakayama, Kyoto University, Sakyo-ku, Kyoto, Japan	N/A
EGFP-ARFIP2 BAR	Zhiqiu Man and Kazuhisa Nakayama, Kyoto University, Sakyo-ku, Kyoto, Japan	N/A
EGFP-ARFIP2 W99	Zhiqiu Man and Kazuhisa Nakayama, Kyoto University, Sakyo-ku, Kyoto, Japan	N/A
EGFP-ARFIP Chimera	This study	N/A
LAMP1-mGFP	Addgene	Cat. #134868
LAMP1-mRFP	Addgene	Cat. #34611
Myc-TurboID	This study	N/A
Myc-ATG9A-TurboID	This study	N/A
AP3-D1	Andrew Peden, School of Bioscience, University of Sheffield, Sheffield, United Kingdom	N/A
mCherry-3xFLAG-ATG9A WT	This study	N/A
mCherry-3xFLAG-ATG9A M8	This study	N/A
mCherry-3xFLAG-ATG9A M33	This study	N/A
TGN46-EGFP	Vas Ponnambalam, Faculty of Biological Sciences, University of Leeds	N/A
EGFP-P4C-SidC	Thomas Balla, Section on Molecular Signal Transduction, National Institutes of Health, Bethesda, MD, USA.	N/A
P4Mx2-SidM-iRFP	Gerald Hammond, Department of Cell Biology at the University of Pittsburgh School of Medicine	N/A
SAC1-GFP	Gerald Hammond, Department of Cell Biology at the University of Pittsburgh School of Medicine	N/A
Software and algorithms		
FIJI (Image J)	Schindelin et al.^83^	https://imagej.net/software/fiji/
GraphPad Prism version 9.5.1 for macOS	N/A	www.graphpad.com
Other		
18:1 DGS-NTA(Ni) 5mg/mL in chloroform	Avanti Polar Lipids	Cat. #790404C
18:1-12:0 NBD–PS (1mg in chloroform)	Avanti Polar Lipids	Cat. #810195C
18:1 Liss Rhod PE	Avanti Polar Lipids	Cat. #810150C
18:1 (Δ9-Cis) PC (DOPC)	Avanti Polar Lipids	Cat. #850375C
DOPE (18:1)	Avanti Polar Lipids	Cat. #850725C
Brain PI4P	Avanti Polar Lipids	Cat. #840045X

## Experimental Model and Study Participant Details

### Cell culture

HEK293A human embryonic kidney cells (CRL-1573), *Mocha* fibroblasts (CRL-2709) and HeLa WT (CCL-2) were obtained from American Type Culture Collection (ATCC). Mouse embryonic fibroblasts (MEF) and SH-SY5Y cells were obtained from Cell Services at The Francis Crick Institute. HeLa Hexa KO cell line was generated previously^38^ and kindly provided by Dr. M. Lazarou.

Cells were grown in DMEM high glucose (Sigma, D6429) supplemented with 10% heat-inactivated fetal bovine serum (FBS) (Gibco, 10270-106). Cells were incubated at 37°C, 10% CO_2_ and 90-95% of relative humidity. Specific experimental conditions are indicated in figure legends. The ARFIP2 KO cell line was generated by CRISPR/Cas9-mediated genome engineering as previously described.^16^ The ATG9A KO cell line was generated by CRISPR/Cas9-mediated genome engineering as previously described.^36^

SH-SY5Y 14-day differentiation protocol was performed as previously described.^75,76^ Briefly, on day 1 and 4, the media was switched to DMEM high glucose supplemented with 2.5% heat-inactivated FBS and 10 μM Retinoic Acid (Merck, R2625). On day 7, cell culture plates were coated with laminin (Corning, 10152421) at 2 μg/cm^2^. On day 8 and 11, the media was switched to a neuron differentiation media consisting of Neurobasal Medium (Thermo Fisher Scientific, 21103049) supplemented with 1x B-27 Supplement (Thermo Fisher Scientific, 17504044), 50 ng/mL Human Recombinant brain-derived neurotrophic factor (BDNF) (Stem Cell Technologies, 78005), 0.2 mM dibutyryl cyclic AMP (db-cAMP) (Merck, 28745), 20 mM Potassium chloride (Sigma-Aldrich, P9531), 2 mM L-Glutamine (Sigma-Aldrich, G7513), and 10 μM Retinoic Acid. On day 14, cells were processed for the specific experiments.

### Preparation and culture of HMDMs

Human monocytes were prepared from leucocyte cones (NC24) supplied by the NHS Blood and Transplant service.^72^ White blood cells were isolated by centrifugation on Ficoll-Paque Premium (17-5442-03; GE Healthcare) for 60 min at 300 × *g*. Mononuclear cells were collected and washed twice with MACS rinsing solution (130-091-222; Miltenyi) to remove platelets and red blood cells. The remaining samples were incubated with 10 ml RBC lysing buffer (R7757; Sigma-Aldrich) per pellet for 10 min at room temperature. Cells were washed with rinsing buffer and were resuspended in 80 μl MACS rinsing solution supplemented with 1% BSA (130-091-376; MACS/BSA; Miltenyi) and 20 μl anti-CD14 magnetic beads (130-050-201; Miltenyi) per 10^8^ cells. After 20 min on ice, cells were washed in MACS/BSA solution and resuspended at a concentration of 10^8^ cells/500 μl in MACS/BSA solution and further passed through an LS column (130-042-401; Miltenyi) in the field of a QuadroMACS separator magnet (130-090-976; Miltenyi). The LS column was washed three times with MACS/BSA solution, then CD14 positive cells were eluted, centrifuged, and resuspended in complete RPMI 1640 with GlutaMAX and Hepes (72400-02; Gibco) and 10% fetal bovine serum (FBS; F7524; Sigma-Aldrich).

### Mycobacterial strains and culture conditions

*Mycobacterium tuberculosis* H37Rv (*Mycobacterium tuberculosis* WT) was kindly provided by Prof. Douglas Young (The Francis Crick Institute, London, UK). Fluorescent *Mycobacterium tuberculosis* strains were generated as previously reported.^77^ E2Crimson *Mycobacterium tuberculosis* was generated by transformation with pTEC19 (30178; Addgene, deposited by Prof. Lalita Ramakrishnan). The strain was verified by sequencing and tested for phthiocerol dimycocerosate positivity by thin-layer chromatography of lipid extracts from *Mycobacterium tuberculosis* cultures. *Mycobacterium tuberculosis* strain was cultured in Middlebrook 7H9 (M0178; Sigma-Aldrich) supplemented with 0.2% glycerol (G/0650/17; Fisher Chemical), 0.05% Tween-80 (P1754; Sigma-Aldrich), and 10% ADC (212352; BD Biosciences).

### *Salmonella* strains and culture conditions

*Salmonella enterica* serovar Typhimurium, strain 14028s were used for all cell culture studies. mCherry-expressing *Salmonella* was grown with 50 μg/mL carbenicillin. 50 μg/mL kanamycin was added for the culture of bacteria.

## Method Details

### Plasmids and siRNA transfection

DNA transfection was performed following manufacturer’s instructions using Lipofectamine 2000 (Invitrogen, 11668-019) in 1:5 Opti-MEM:DMEM medium (Gibco, 31985-047). Plasmids generated for this study are available upon request: pcDNA3.1-mCherry-3xFLAG-His-TEV-ATG9A, pcDNA3.1-myc-TurboID, pcDNA3.1-myc-ATG9A-TurboID. pBMN-AP3D1 was kindly provided by Dr A. Peden.^48^ pGEX6p-1 GST-ARFIP2, pDEST-GFP-ARFIP2 and GFP-BAR were a gift from K. Nakayama^24^ (Kyoto University, Kyoto, Japan) and GFP-ARFIPs Chimera was generated from pDEST-GFP-ARFIP2. PI4K2A plasmids were a gift from Shane Minogue (UCL, Institute for Liver and Digestive Health, London, United Kingdom). pEGFP-N1 TGN46-GFP was a gift from V Ponnambalam (University of Leeds, United Kingdom). pcDNA3.1-mCherry-3× FLAG-6× His-TEV-ATG9A was purchased from Genescript. GFP-SACM1L and GFP-P4MX2 were kindly gifted from G. Hammond^78^ (University of Pittsburgh, Pittsburgh, PA). EGFP-P4C-SidC plasmid was a gift from Michael Marks (University of Pennsylvania, Philadelphia, USA) and Tamas Balla (NIH, Bethesda, USA). GFP-VAPB plasmid was a gift from Tim Levine (UCL Institute of Ophthalmology, London, United Kingdom). pC3 OSBPL9 and OSBPL11 plasmids were kindly gifted by JX Tan^6^ (Aging Institute, University of Pittsburgh School of Medicine and University of Pitts-burgh Medical Center, Pittsburgh, PA, USA).

### Nucleofection of HMDM

Human monocytes were washed twice with PBS and electroporated in the appropriate primary nucleofection solution (Cat. No. VPA-1007; Amaxa Human Monocyte Nucleofector Kit) using the Lonza 2b Nucleofector (AAB-1001; Nucleofector 2b Device). 5 × 10^6^ of human monocytes were used per reaction and resuspended in 100 μl of primary nucleofection solution containing 4 μg of S.p. Cas9 (IDT) mixed with a total of 12 μg of targeting synthetic chemically modified sgRNAs (Synthego). Human monocytes were then nucle-ofected with the sgRNA pool and the Cas9-RNP mix using the Y001 program. Nucleofected cells were cultured in prewarmed RPMI 1640 supplemented with GlutaMAX, Hepes, and 10% FBS in a 6-well plate. 2 h after nucleofection, 100 ng/ml hM-CSF was added to the cells. Dishes were incubated in a humidified 37°C incubator with 5% CO_2_. After 3 d, an equal volume of fresh complete media including 100 ng/ml hM-CSF was added. 6 d after the initial isolation, differentiated macrophages were detached in 0.5 mM EDTA in ice-cold PBS using cell scrapers (83.1830; Sarsted), pelleted by centrifugation, and resuspended in RPMI medium containing 10% FBS.^79^

### Macrophage infection with *Mycobacterium tuberculosis*

The day before infection, HMDM were seeded at a density of 60,000 cells per well of a 96-well plate. Mid-logarithmic phase bacterial cultures (OD_600_ 0.5-1.0) were centrifuged at 2,000 × *g* for 5 min and washed twice in PBS. Pellets were then shaken vigorously for 1 min with 2.5–3.5 mm glass beads (332124G; VWR) and bacteria were resuspended in 10 ml macrophage culture media before being centrifuged at 300 × *g* for 5 min to remove large clumps. The top 7 ml of bacterial suspension was taken, OD_600_ recorded and diluted appropriately for infection. The inoculum was added at the correct MOI, assuming OD_600_ of 1 is 1 × 10^8^ bacteria/ml. Infections were carried out in a volume of 50 μl in a 96-well plate, 300 μl in a 24-well plate, or 500 μl in a 12-well plate. After 2 h of uptake, extracellular bacteria were removed with two washes in PBS and macrophages were incubated at 37°C and 5% CO_2_ for the required time points in macrophage media. An MOI of 1 was used for replication experiments.

### Long-term live-cell imaging of *Mycobacterium tuberculosis* replication and HMDM

For live-cell imaging, 60,000 macrophages were seeded per well on an olefin-bottomed 96-well plate (6055302; Perkin Elmer). Cells were infected with *Mycobacterium tuberculosis* at an MOI of 1 for 2 h. After infection, cells were washed with PBS and replaced with a macrophage media. Imaging was performed using the OPERA Phenix microscope with a 40× 1.1 NA water-immersion objective with a 10% overlap between adjacent fields. Five planes with 1 μm distance of more than 20 fields of view were monitored in time and snapshots were taken every 1.5 h for 72 h. For imaging on the Opera Phenix, Brightfield was detected using λex = transmission/λem = 650–760 nm, and E2-Crimson bacteria was detected using λex = 640 nm/λem = 650–760 nm using a 16-bit scMOS camera. For assessing bacterial replication, analyses were performed with Harmony software where maximum projection of individual z-planes with an approximate distance of 1 μm was used. To perform cellular segmentation “Find texture regions,” building blocks were trained in Brightfield channel to segment cellular areas. Following the segmentation of cellular area Find spots, building blocks were used to segment *Mycobacterium tuberculosis*. To determine the bacteria area over time, the spot area was summed for each time point. *Mycobacterium tuberculosis* replication as growth index was calculated by the formula: (sum of intracellular *Mycobacterium tuberculosis* area for the time point - sum of intracellular *Mycobacterium tuberculosis* area t0 h) / (sum of intracellular *Mycobacterium tuberculosis* area t0 h).

### *Salmonella* infection

For SPI-1 induced infection of HEK293A cells, bacteria were grown overnight in Luria broth (LB) and sub-cultured (1:33) in fresh LB for 3.5 h prior to infection at 37 °C. Cells seeded in 24-well were infected with 10 μL of *Salmonella* subculture, for 10 min at 37 °C. After two PBS washes, cells were incubated in 100 μg/mL gentamycin for 1-2 h and 20 μg/mL gentamycin thereafter.

### Proximity labelling using TurboID and streptavidin pull-down

Stable myc-TurboID and myc-ATG9A-TurboID HEK293A cells were cultures in 15 cm Petri dishes until they reached 80% confluency. Cells were treated with 50 μM Biotin in complete medium to allow biotinylation at different time points (1 hr for MS experiments and 15 minutes for Western Blot validation). After Biotin treatment, cells were washed 3 times with ice-cold PBS and centrifuged to generate cell pellets that were frozen. For Western Blot analysis, lysates were lysed in 700 μL TNTE lysis buffer [20 mM Tris-HCl pH 7.4, 5 mM EDTA, 150 mM NaCl, 0.5% Triton-X100] supplemented with EDTA-free protease (Roche, cOmplete EDTA-free, 05056489001) and phosphatase inhibitors (Roche, Phostop EASYpack, 04 906 837 001). Cell lysates were centrifuged at 13000 x g for 10 min at 4 °C. Lysates were pre-cleared with 15 μL of empty agarose beads in a wheel for 1h at 4°C. Protein concentration was determined by Bradford (Bio-Rad, Bio-Rad Protein Assay Dye Reagent Concentrate, 5000006) and protein amounts were normalized among samples. Inputs 2% were taken for Western Blot analysis. Pre-cleared lysates were subjected to Streptavidin pull-down by incubating samples with 30 μL washed beads in a wheel for 2h at 4°C supplemented with 1% SDS. After pull-down, beads were washed three times (10 minutes each) at 4°C and eluted at 65°C for 15 minutes in elution buffer (Laemmli buffer + 3 mM Biotin). Samples were further processed for either Western Blot or label-free MS as described in the corresponding sections.

### GFP-trap immunoprecipitation

Cells were lysed in ice-cold TNTE buffer (20 mM Tris, pH 7.4, 150 mM NaCl, 1%w/v TritonX-100, 5 mM EDTA) containing EDTA-free Complete Protease Inhibitor cocktail (Roche). Lysates were cleared by centrifugation at 21000 x g and precleared with binding control agarose beads (ChromoTek) for 1 hour at 4°C. GFP-tagged proteins were immunoprecipitated using GFP-TRAP beads (ChromoTek) for 2h at 4 °C. After immunoprecipitation, GFP-beads were washed three times with ice-cold TNTE buffer and resuspended in 2X Laemmli sample buffer. Samples were further processed for either Western Blot or label-free MS as described in the corresponding sections.

### Cell lysis and Western Blot

For cell lysis, cells were washed three times with ice-cold PBS, scraped and lysed on Lysis Buffer [20 mM Tris-HCl pH 7.4, 5 mM EDTA, 150 mM NaCl, 0.5% Triton-X100] supplemented with EDTA-free protease (Roche, cOmplete EDTA-free, 05056489001) and phosphatase inhibitors (Roche, Phostop EASYpack, 04 906 837 001). Cell lysates were centrifuged at 13000 x g for 10 min at 4°C. Protein concentration was analyzed using Pierce BCA Protein Assay kit (Thermo Scientific, 23227) following manufacturer’s instructions. Equal amounts of protein lysates were resuspended in Laemmli SDS-sample buffer and incubated at 65°C for 10 min. Proteins were resolved on NuPage 4-12% Bis-Tris Gel (Invitrogen, NP0336) using MES or MOPS SDS running buffer (Invitrogen, NP0002 or NP0001) and transferred to Immobilon-P transfer membranes (Millipore, IPVH00010). Membranes were blocked with 5% non-fat dry milk (BioRad, 1706404) in Phosphate-buffered saline containing 0.1% Tween-20 (PBS-T) for 1 h at room temperature. Incubation of primary antibodies was performed overnight at 4°C in 5% non-fat dry milk or 3.5% BSA (Roche, 10735086001). After three washes in PBS-T, membranes were incubated for 1 h at room temperature with secondary antibodies (1:5000) diluted in 5% non-fat milk. Upon incubation, membranes were washed three times with PBS-T and protein detection was performed by using Immobilon Classico Western HRP Substrate (Millipore, WBLUC0500) or Immobilon Crescendo Western HRP Substrate (Millipore, WBLUR0500). Blots were scanned with Amersham ImageQuant 800 (Cytiva). Densitometry analysis of Western Blots was performed using FIJI (https://fiji.sc/). All the antibodies used in this study are reported in key resources table.

### Mass spectrometry LC-MS/MS and MS data processing and analysis

For the Proximity labelling assay using TurboID and streptavidin pull-down MS experiments were performed by DDA analysis on Orbitrap Fusion Lumos. Peptides were analyzed using an Evosep One LC system (EvoSep Biosystems) directly coupled to an Orbitrap Fusion Lumos tribrid mass spectrometer (Thermo Scientific). Reverse phase separations were performed at a flow rate of 500 nL/min on an EV1064 ENDURANCE analytical column (100 μm × 8 cm, 3.0 μm particle size; Evosep Biosystems) using the vendor’s predefined 30 samples per day gradient method. The Orbitrap was operated in ‘TopS’ Data Dependent Acquisition mode with precursor ion spectra acquired at 120k resolution in the Orbitrap detector and MS/MS spectra at 32% HCD collision energy in in the ion trap. Automatic Gain Control was set to Auto for MS1 and MS2. Maximum injection times were set to ‘Standard’ (MS1) and ‘Dynamic’ (MS2). Dynamic exclusion was set to 20s. For the DDA Data Processing and Analysis MaxQuant (version 1.6.12.0) was used for data processing. The data was searched against the *Homo Sapiens* UniProt reference proteome. A decoy database containing reverse sequences was used to estimate false discovery rates and set the false discovery rate at 1%. Default MaxQuant parameters were used with the following adjustments: Label-free quantification was selected along with iBAQ values, with Normalization type “Classic” selected. MaxQuant output files were imported into Perseus (version 1.4.0.2) and the LFQ intensities and iBAQ values were used for all subsequent analysis. Missing values were imputed from a normal distribution.

For the GFP-trap immunoprecipitation experiments, MS experiments were performed by Data-independent acquisition (DIA) analysis on Fusion Lumos Orbitrap. Peptides were analyzed using an Evosep One LC system (EvoSep Biosystems) directly coupled to an Orbitrap Fusion Lumos tribrid mass spectrometer (Thermo Scientific). Reverse phase separations were performed at a flow rate of 500 nL/min on an EV1064 ENDURANCE analytical column (100 μm × 8 cm, 3.0 μm particle size; Evosep Biosystems) using the vendor’s predefined 30 samples per day gradient method. Lumos instrument settings were as follows: MS1 data acquired in the Orbitrap with a resolution of 120 k, max injection time of 20 ms, AGC target of 1e6, in positive ion mode, in profile mode, over the mass range of 393 to 907 *m*/*z*. DIA segments over this mass range (20 *m*/*z* wide/1 Da overlap/27 in total) were acquired in the Orbitrap following fragmentation in the HCD cell (32%), with 30 k resolution over the mass range 200 to 2,000 *m*/*z* and with a max injection time of 54 ms and AGC target of 1e6. For the DIA-MS Data Processing and Analysis, the data were searched using Direct DIA data analysis on Spectronaut v.14 (Biognosys AG) using default settings, then run-wise imputation (*Q*-value percentile = 30%) were applied to the dataset. A two-sample *t*-test was carried out in Spectronaut software, then filters were applied to the data (*q* ≤ 0.05, avg log_2_-fold change ≥ 0.58, no. unique total peptides ≥ 2). The mass spectrometry proteomics data have been deposited to the ProteomeXchange Consortium via the PRIDE^80^ partner repository with the dataset identifiers PXD056417 and PXD056398.

### Immunofluorescence and confocal microscopy

Cells were grown on poly-D-lysine treated coverslips at a 70% confluency the day of the experiments. After the treatments, cells were fixed with 4% paraformaldehyde in phosphate-buffered saline (PBS) supplemented with 0.1 mM CaCl_2_, 0.1 mM MgCl_2_ for 10 min. Cells were washed three times with PBS before adding 50 mM NH_4_Cl for 10 min at room temperature and then permeabilized with 50 μg/mL digitonin (Merck Millipore; D141) for 5 min at room temperature. Coverslips were then washed three times with PBS before the addition of the blocking solution (5% BSA in PBS) for 30 min at room temperature. Coverslips were incubated with primary antibody diluted in 1% BSA/PBS for 1 h at room temperature, then washed three times with PBS and incubated with secondary antibody in 1% BSA/PBS for 1 h. Finally, coverslips were washed three times in PBS and once with demineralized water before mounting them on glass microscope slides with 10 μL Mowiol mounting solution per coverslip. Fluorescence images were acquired using a Zeiss LSM 880 Airyscan confocal microscope with Plan-Apochromat 63x/1.4 Oil DIC M27 objective lens. Zeiss ZEN imaging software was used for the acquisition. Antibodies used in this study are listed in in key resources table. For SoRa imaging, images were acquired on a Nikon SoRa spinning disk, using a 60X/1.4 Oil immersion objective and SoRa magnification disk to get a final pixel size of 27 nm x 27 nm. The different channels were acquired exciting with the laser lines 405 nm, 488 nm, and 561 nm, and detecting with selective bandpass filters for DAPI (447/60 nm), Alexa Fluor 488 (525/50 nm), and Alexa Fluor 555 (600/52 nm).

### Immunogold labelling and electron microscopy methods

Immunogold labeling of thawed cryosections was performed as described.^81,82^ In brief, cells were fixed for 2h with 4% FA in PHEM buffer (EGTA, HEPES, MgCl_2_6H_2_O, NaOH, PIPES), washed and scraped before being pelleted and embedded in 12% (w/v) gelatin. Embedded cell pellets were cut into smaller (0.5 mm^3^) blocks, mounted on aluminium pins and snap frozen in LN2. After sectioning at circa -100°C, grids were incubated at 37°C for 30 min, washed with PBS 0.15% glycine, blocked and labelled using primary antibodies for 1h at RT. In case of single labelling, rabbit-anti-ATG9A STO219 was followed by protein A-gold-10 nm for 20 min and then fixation in 1%GA for 3min. In case of double labelling, Rabbit-anti-ATG9A STO219 was followed by protein A-gold-15nm, fixed with 1%GA for 3 min and followed by mouse-anti-LAMP1 (BD Pharmingen, 555798) and a bridging antibody rabbit-anti-mouse (Rockland, 610-4120) followed by protein-A-gold-10 nm and fixation with 1% GA. Grids were then washed extensively before staining with Uranyl Acetate. Immuno-EM samples were imaged on a Tecnai T12 TEM (FEI Tecnai) using SerialEM or Radius software.

### Lysotracker uptake and recovery

Twenty thousand cells were seeded into 96-well plates (Greiner Bio One Ltd 655090). Cells were loaded with the nuclear dye Hoechst 33342 (Thermo Fisher Scientific) at a dilution of 1:10,000 and 25 nM LysoTracker DND-99 (Thermo Fisher Scientific; L7528) for 45 minutes. The cells were imaged every 1 min at 37 °C, 5% CO2 using an Opera Phenix microscope (PerkinElmer). First, a baseline was established by imaging 3 time points corresponding to 20 minutes, followed by the addition of LLOMe to a final concentration of 1 mM. After 15 minutes, the cells were washed 3 times with DMEM and the medium was replaced with DMEM containing 25 nM LysoTracker, and lysosomal recovery was followed for 80 min. For the Lysotracker uptake assay, the microscope was pre-warmed at 37°C and supplied with 5% CO2, prior to imaging. Cells were imaged with 63x/NA (1.15) water-immersion lens. 4 Z-stacks with a step size of 1μm were imaged were acquired using excitation lasers at 375, and 568 nm, and emission filters at 435-480, and 570-630 nm, respectively. Cell segmentation and quantification analysis were performed using Harmony software 5.0.

### Live imaging

Cells were seeded on glass-bottom microwell dishes (MatTek Corp.; P35G-1.5-14-C) to reach a 70% confluency the day of the experiment. For LysoTracker uptake, transfected cells were loaded with Lysotracker Blue DND-26 (Thermo Fisher Scientific; L7525) diluted in full medium according to manufacturer’s instructions. Cells were imaged using a Zeiss LSM 880 Airyscan confocal microscope with Plan-Apochromat 63x/1.4 Oil DIC M27 objective lens. Live imaging was performed using Zeiss ZEN imaging software. After the acquisition, movies were processed using an Airyscan processing tool on the ZEN software provided by Zeiss.

### Image analysis

Image analysis has been performed using the open-source FIJI (http://fiji.sc)^83^ and the pipelines for the different quantified phenotypes have been designed as follows: (i) Overlap of proteins with a specific organelle (Golgi or Lysosome). The protein of interest was used to threshold and generate a binary image of total cytoplasmic fluorescence. The organelle marker (GM130, TGN46, LAMP1 or Lysotracker) was used to generate a mask of the organelle region. The ratio between the mean intensity of Protein of interest in the masked organelle / total mean intensity was calculated as previously described,^50^ (ii) ATG9A intracellular dispersal. In cases where a Golgi marker was not used, a mask of the nuclei was used to create a distance map corresponding to the segmented cells. ATG9A intensity was calculated according to the distance map to estimate the dispersal from the nuclei upon starvation, (iii) LGALS3 spot counting. LGALS3 channel was used to create a binary image that reflected the positive signal by adjusting the threshold. The “analyze particles” plugin in FIJI was used to determine the number. Nuclei counting was used to normalize the number of spots per cell, (iv) *Salmonella*/*Mycobacterium tuberculosis* area per cell. *Salmonella* or *Mycobacterium tuberculosis* were used to mask and create a binary image to calculate their respective total area that was then normalized on the number of nuclei per image (v) ATG9A/PI4K2A localization in *Salmonella. Salmonella* was used to mask and create a binary image to calculate the ratio of ATG9A or PI4K2A on the particle versus total ATG9A or PI4K2A.

### Immunoisolation of ATG9A-positive membranes

ATG9A-positive membranes were isolated by adapting the protocol established in our laboratory.^16^ Briefly, cells were washed in ice-cold PBS and harvested by centrifugation at 200 x g at 4 °C. Pellets were resuspended using ice-cold isotonic buffer (20 mM HEPES, pH 7.4, 250 mM sucrose, and 1 mM EDTA) supplemented with EDTA-free complete protease and phosphatase inhibitors. The resuspended pellet was passed through a 27G needle 15 times for homogenization before clarification by centrifugation at 3,000 x g at 4 °C. Supernatants were incubated overnight at 4 °C with hamster anti-ATG9A or hamster IgM CTRL coupled with protein A Dynabeads (Invitrogen; 10002D). The ATG9A-positive membranes were washed three times with isotonic buffer at 4 °C and resuspended in 2× Laemmli sample buffer before being resolved by SDS-PAGE and Western blotting.

### Lysosome immunoprecipitation

LAMP1-positive membranes were isolated by adapting a protocol previously established.^84^ Briefly, cells were washed in ice-cold PBS and harvested by centrifugation at 200 x g at 4 °C. Pellets were resuspended in SuMa^4^ buffer (10 mM HEPES, 70 mM sucrose, 210 mM mannitol) that was supplemented with 0.5 mM DTT, 25 mg/mL fatty acid-free BSA, a tablet of Mini Complete protease inhibitor without EDTA (Sigma-Aldrich, #5056489001), and ≤ 5 units benzonase (Sigma-Aldrich, #E1014-25KU)), resuspended and lysed in 1 mL SuMa^4^ buffer by syringing the cells through a 25-gauge needle. This is referred to as “whole cell sample/fraction”. The lysates were centrifuged at 1000× g for 10 min, and the supernatant was transferred to a new tube, and the step was repeated. Following, the lysates were mixed with either 0.6 μg/mL IgG control (Invitrogen, Waltham, MA, USA, #31235) or anti-LAMP1 primary antibody (Abcam, #ab24170) coupled with protein A Dynabeads (Invitrogen; 10002D) and incubated for 30 min in a rotator at 4 °C. The ATG9A-positive membranes were washed in SuMa^4^ once and three times in SuMa^2^ buffer (SuMa4 buffer without benzonase and a protease inhibitor tablet) and resuspended in 2× Laemmli sample buffer before being resolved by SDS-PAGE and Western blotting.

### Differential centrifugation for cell fractionation

Cells were seeded in two 15 cm dishes per condition to reach 80% confluency the day of the experiment. Cell monolayers were washed twice with ice-cold PBS and scraped in 5 ml PBS followed by centrifugation at 100 x g for 5 minutes to obtain the cell pellet. Cell pellet was resuspended in 700 uL of ice-cold isotonic buffer (20 mM HEPES, pH 7.4, 250 mM sucrose and 1 mM EDTA) supplemented with EDTA-free protease (Roche, cOmplete EDTA-free, 05056489001) and phosphatase inhibitors (Roche, Phostop EASYpack, 04 906 837 001). Cells were mechanically lysed by passing them through a 27G needle attached to a 1 mL syringe on ice. To lyse MEFs, 25 up and down passes were needed. The protocol was adapted from Shoemaker et al., 2019.^85^ The post-nuclear supernatant (PNS) was obtained by centrifugation at 1000 x g for 10 minutes at 4 °C to remove nuclei and cell debris. Differential centrifugation protocol using the following speeds were subsequently performed: 3000 x g for 20 minutes, 20000 x g for 30 minutes and 100000 x g for 30 minutes. Fractions 1 and 2 were treated with Benzonase to eliminate any DNA traces and fraction samples were run in SDS-PAGE as described above.

### Protein expression and purification

ARFIP2 (pGEX-6P-1-GST) was transformed in Escherichia coli BL21 (DE3) cells. Bacterial were grown at 37°C in LB to an OD600 of 0.6-0.8. Protein expression was induced with 0.5 mM IPTG for 16 hrs at 18°C. Cells were harvested by centrifugation and resuspended in ice cold lysis buffer containing 50 mM Tris-HCl pH 7.5, 500 mM NaCl, 0.5 mM TCEP, 0.4 mM AEBSF, and 15 μg/ml benzamidine. Cells were lysed by freeze-thaw, followed by sonication. Cell lysate was cleared by centrifugation at 25,000g for 30min, at 4°C. The protein was absorbed onto 1mL of Glutathione-Sepharose 4B affinity matrix (GE Healthcare) for 2 hrs and recovered by homemade 3C protease cleavage at 4°C overnight in 50 mM Tris-HCl pH 7.5, 500 mM NaCl, and 0.5 mM TCEP. The eluted protein was further purified by size-exclusion chromatography using Superdex 200 16/60 column (GE Healthcare) equilibrated in buffer containing 25 mM Tris-HCl pH 7.5, 150 mM NaCl, 0.5 mM TCEP and 5% glycerol.

Flag-OSBPL9/11 heterodimer was expressed and purified from Expi293 cells (ThermoFisher). To transfect cells, cells were grown to a density of 2-3×10^6^/mL on a shaker at 130 rpm, at 37°C, supplemented with 8% CO_2_ in Expi293 Expression medium. 1μg of plasmids (OSBPL9: OSBPL11, in amount ratio 1:1) per 1×10^6^ cells was mixed with a threefold (w/w) of PEI (polyethyleneimine, linear MW 25000, Polysciences) in Opti-MEM. The transfection mixture was added to the cells, and the cells were grown for another 72 hrs. The transfected cells were pelleted and resuspended in the lysis buffer containing 50 mM Tris-HCl pH 8.0, 500 mM NaCl, 0.5 mM TCEP, 5% glycerol, EDTA-free Complete Protease Inhibitor cocktail (Roche) and 0.1% Triton X-100. Cells were disrupted by three times freeze-thaw cycles in liquid nitrogen and water bath. The cell lysate was cleared by centrifugation at 20,000g, for 30min at 4°C. The supernatant was incubated with anti-DYKDDDDK G1 affinity resin (Genscript) for 4-5 hours at 4°C. The resin was washed with buffer containing 50 mM Tris-HCl pH8.0, 500 mM NaCl, 0.5 mM TCEP, 5% glycerol and the proteins were eluted with 5 mg/mL 3xFlag peptide dissolved in water. The eluted protein was further concentrated by 30kDa molecular weight cut-off centrifugal concentrator, snap frozen in liquid nitrogen, stored at -80 °C.

### Liposome preparation and Lipid transfer assay

Lipids were mixed at the indicated molar ratio in chloroform, dried to a lipid film under nitrogen gas, and further vacuumed for 2 hrs. The lipid film was rehydrated and resuspended in the assay buffer containing 25 mM Tris-HCl pH 8.0, 150 mM NaCl and 0.5 mM TCEP, with vortexing. The resuspended lipid solution was subjected to 5 cycles of freeze-thaw in liquid nitrogen and water batch. The liposome solution was extruded 10 times through 0.2 μm membrane, followed by at least 20 times through 0.1 μm membrane via a Mini-Extruder (Avanti Polar Lipid). The final concentration of liposomes was 1 mM. The liposomes had an average diameter of 100 nm, measured by Zetasizer Nano ZS (Malvern Instruments).

Lipid transfer assays were performed at 25°C with at least three independent repeats. Donor liposomes contained 66% DOPC, 25% DOPE, 5% DGS-NTA (Ni), 2% NBD-PS and 2% Rh-PE. Acceptor liposomes contained 75% DOPC and 25% DOPE, or 70% DOPC, 25% DOPE and 5% brain PI4P. Briefly, the reaction sample (final volume: 80 μL) containing 100 nM OSBPL9/11 (heterodimer), 20 nM or 50 nM ARFIP2 was mixed with 100μM donor liposomes and 100 μM acceptor liposomes. The reaction mix was then transferred into 10 mm pathlength quartz cuvette and the NBD fluorescence (excitation at 468 nm, emission at 535 nm) was recorded using FP-8300 spectrofluorometer (JASCO), every 40 sec. Total measurement time was set to 3240 sec. The excitation bandwidth was fixed to 5 nm, and the emission bandwidth was fixed to 10 nm. As a background control, the reaction sample containing only 100 μM donor liposomes was recorded (“donor only”). The fluorescence increase (ΔEm535 nm) at each time point was calculated by subtracting the NBD signal recorded from the “donor only” group.

## Quantification and Statistical Analysis

Statistical analyses were performed using GraphPad Prism version 9.5.1 for macOS, GraphPad Software, San Diego, California, USA (www.graphpad.com) according to their recommendations. Normality of the data as well as SD similarity were tested before any statistical test for differences was performed. For two sample comparisons, an unpaired two-sided t-test was used for Gaussian-distributed data with similar SD, while Welch correction was applied for normal data with different SD distribution. In cases where Gaussian distribution could not be assumed, the Mann-Whitney test for rank comparisons was performed to determine significance. For more than 2 sample comparisons, ordinary One-way ANOVA followed by Dunnett’s multiple comparisons test was used when data followed Gaussian distribution and presented equal variance. In normal-distributed datasets where SD was different, Brown-Forsythe and Welch ANOVA tests were performed followed by Dunnett’s T3 multiple comparisons test. For data that did not pass the normality tests, statistical significance was calculated using the Kruskal-Wallis test followed by Dunn’s multiple comparisons tests. Significance is noted as: ns > 0.05, * p < 0.05, ** p < 0.005, *** p < 0.001, **** p < 0.0001 and graphs represent mean ± SD unless otherwise specified in the figure legend.

## Supplementary Material

Supplemental information can be found online at https://doi.org/10.1016/j.devcel.2025.05.007.

Video S1

Video S2

Video S3

Video S4

Figure S1

Table S1

Table S2

## Data Availability

All data supporting the findings of this study are available in the main text or the supplemental information. The mass spectrometry proteomics data have been deposited to the ProteomeXchange Consortium via the PRIDE partner repository with the dataset identifiers PRIDE: PXD056417 and PXD056398. Any additional information required to reanalyze the data reported in this paper is available from the lead contact upon request.

## In brief

De Tito, Almacellas, et al. discover that ATG9A vesicles deliver PI4K2A to damaged lysosomes, promoting PI4P production for membrane repair. ARFIP2, a PI4P-binding protein, coordinates repair by regulating PI4P availability, lipid transfer, and AP-3-mediated ATG9A and PI4K2A retrieval, highlighting a finely tuned mechanism crucial for lysosomal integrity upon damage or infection.

## Highlights

- ATG9A vesicles deliver PI4K2A to damaged lysosomes to produce PI4P for repair
- ARFIP2 controls OSBPLs-mediated lipid transfer on the lysosomes by binding PI4P
- AP-3 retrieves ATG9A and PI4K2A from the endolysosomal compartment
- Loss of ARFIP2 enhances resistance to *Salmonella* and *Mycobacterium tuberculosis* infection
