# Supplementary material for: ATG9A and ARFIP2 cooperate to control PI4P levels for lysosomal repair
Source: Dev Cell. Author manuscript; Available in PMC 2025 Jul 4. (PMC7617826; doi:10.1016/j.devcel.2025.05.007)
Supplement: Figure S1 [file EMS206349-supplement-Figure_S1.pdf]

Developmental Cell, Volume *60*

Supplemental information

ATG9A and ARFIP2 cooperate to control PI4P levels

for lysosomal repair

Stefano De Tito, Eugenia Almacellas, Daniel Dai Yu, Emily Millard, Wenxin Zhang, Cecilia de Heus, Christophe Queval, Javier H. Hervás, Enrica Pellegrino, Ioanna Panagi, Ditte Fogde, Teresa L. M. Thurston, Judith Klumperman, Maximiliano Gutierrez, and Sharon A. Tooze

1 **Supplementary Figures**

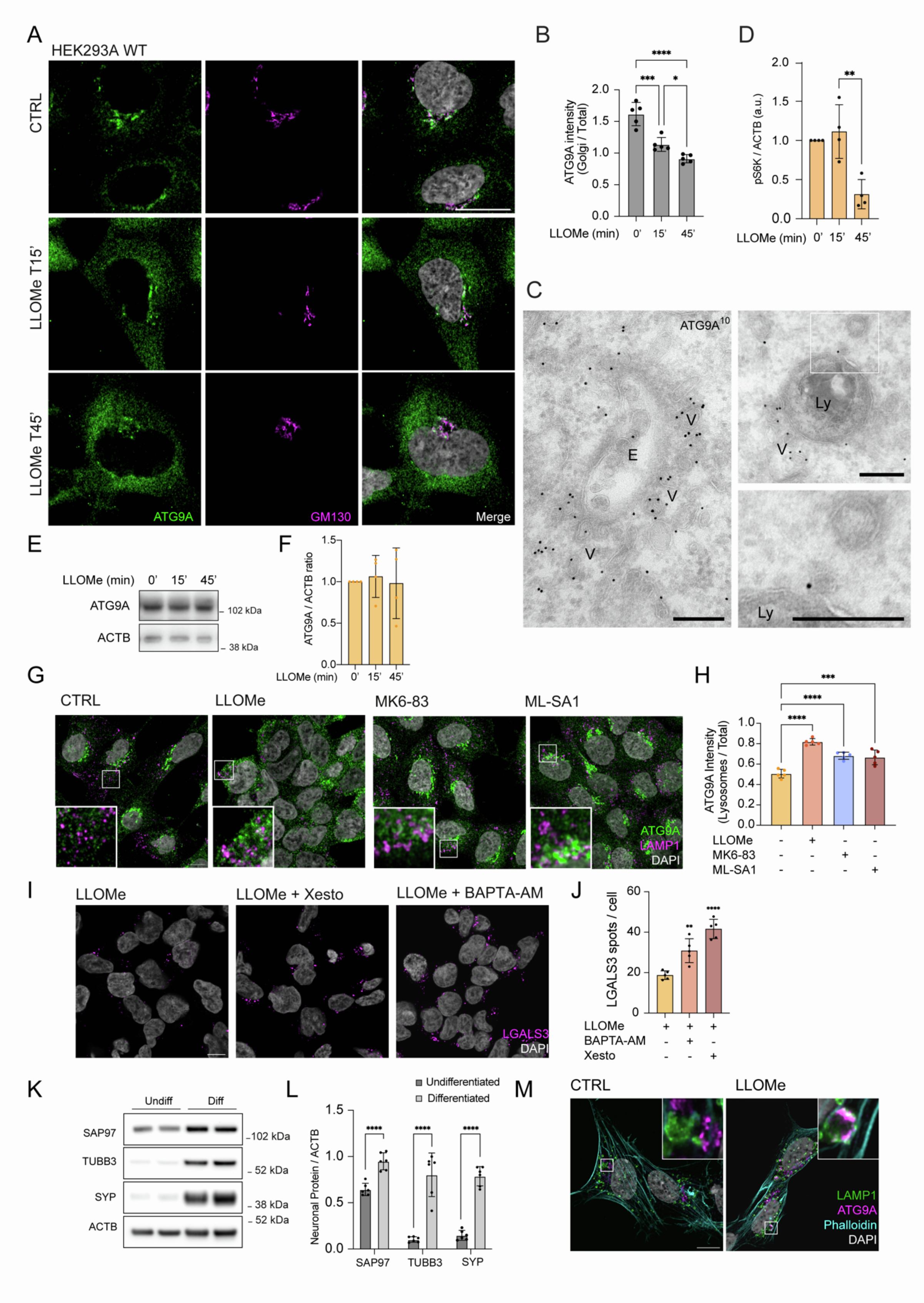

Figure S1. ATG9A disperses and relocalizes on lysosomes upon lysosomal damage but is not degraded, related to Figure 1. (A) HEK293A WT cells were treated with 1 mM LLOMe for the indicated times followed by immunofluorescence using anti-ATG9A and anti-LAMP1 antibodies and DAPI for nuclear staining. Scale bar: 10 µm. (B) Quantification of ATG9A on the Golgi. $n = 5$ independent experiments, * $p < 0.05$, *** $p < 0.001$, **** $p < 0.0001$. (C) Electron micrographs of LLOMe treated cells immunogold labelled for ATG9A (10 nm gold). ATG9A is predominantly found in small vesicles and occasionally on the limiting membrane or in the lumen of endosomes (E) and lysosomes (Ly). The zoom in shows an ATG9A labelled vesicle in very close proximity of the lysosomal membrane. Scale bars: 200 nm. (D) Quantification of phospho-S6-Kinase intensity. $n = 4$ independent experiments, ** $p < 0.01$. (E) HEK293A CTRL cells were treated with 1 mM LLOMe for the indicated times and Western Blot was performed. (F) Quantification of ATG9A intensity from (D). $n = 4$ independent experiments. (G) Cells were treated with TRPML1 agonists (MK6-83, 25 µM and ML-SA1, 25 µM) for 15 minutes prior to the addition of LLOMe 1 mM for 15 minutes followed by immunofluorescence experiments using anti-ATG9A and anti-LAMP1 antibodies (H) Quantification of (F), $n = 5$, *** $p < 0.001$, **** $p < 0.0001$. (I) HEK293A were treated or not with BAPTA-AM (50 µM) or Xestospongin C (10 µM) for 30 minutes before treatment with LLOMe for 15 minutes followed by immunofluorescence to detect LGALS3 spots. (J) Quantification of LGALS3 spots from (I). (K) SH-SY5Y cells were differentiated as described. Undifferentiated (undiff) and differentiated (diff) cells were analyzed by Western Blot with neuronal markers. (L) Quantification of (J) shows an increase in differentiation markers. $n = 6$ independent experiments, **** $p < 0.0001$. (M) Differentiated SH-SY5Y cells were treated with LLOMe 1 mM for 15 minutes and subjected to immunofluorescence analysis using Phalloidin, anti-ATG9A anti-LAMP1 antibodies. Scale bar: 10 µm.

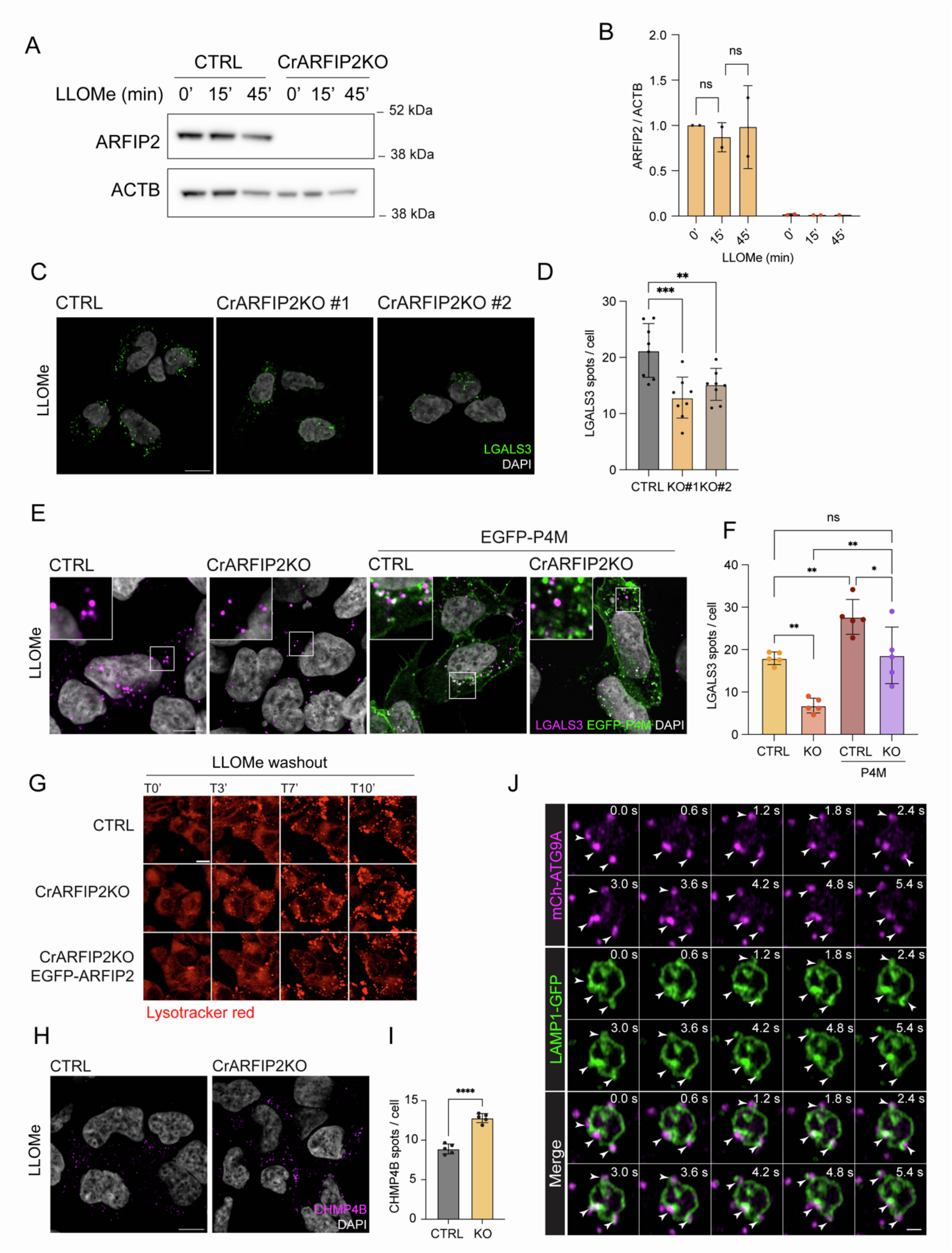

Figure S2. ARFIP2 regulates lysosomal repair through ATG9A, related to Figure 2. (A) HEK293A CTRL and CrARFIP2KO cells were treated with 1 mM LLOMe for the indicated times and ARFIP2 protein levels were analyzed by Western Blot. (B) Quantification of ARFIP2 levels from (A). *n* = *2* independent experiments. (C) HEK293A CTRL and

CrARFIP2KO clone #1 and clone #2 were treated with LLOMe 1 mM for 15 minutes followed by immunofluorescence using anti-LGALS3 antibody. Scale bar: 10 μm. (D) Quantification of (C). $n=2$ independent experiments, ** $p < 0.01$, *** $p < 0.001$. (E) CTRL and CrARFIP2KO cells were transfected with EGFP-P4M and treated with LLOMe for 15 minutes followed by immunofluorescence to detect LGALS3 (F) Quantification of LGALS3 spots from (E), $n = 5$, ** $p < 0.01$, * $p < 0.05$. (G) Representative images of Lysotracker recovery after washout in HEK293A CTRL, CrARFIP2KO and CrARFIP2KO cells expressing EGFP-ARFIP2 cell lines at different time point (minutes). (H) CTRL and CrARFIP2KO cells treated with LLOMe for 15 minutes were subjected to immunofluorescence using anti-CHMP4B antibody. Scale bar: 10 μm. (I) Quantification from (H), $n = 5$, **** $p < 0.0001$. (J) Frames of live imaging experiments, at the indicated time points, of CrARFIP2KO cells transfected with LAMP1-GFP and mCherry-3xFLAG-ATG9A. Arrowheads indicate the overlap between ATG9A and LAMP1. Scale bar: 1 μm.

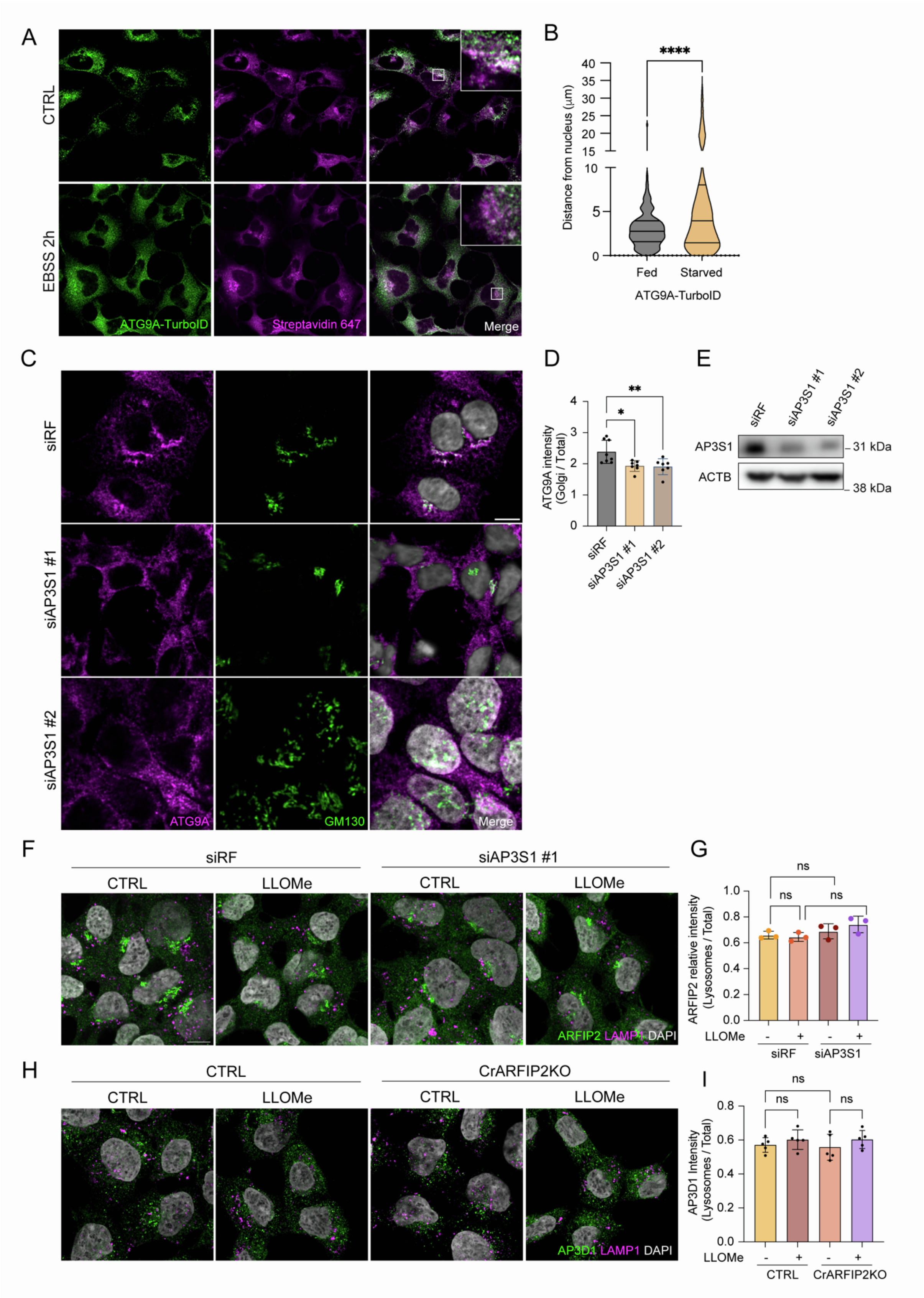

Figure S3. AP-3 controls ATG9A trafficking throughout the endolysosomal compartment, related to Figure 3. (A) HEK293A expressing myc-ATG9A-TurboID were

subjected to starvation (EBSS 2h) and ATG9A dispersal was detected by immunofluorescence using anti-ATG9A antibody, Streptavidin A647 and DAPI for nuclear staining. Scale bar: 10 µm (B) Quantification of ATG9A dispersal upon starvation. $n = 3$ independent experiments, **** $p < 0.0001$. (C) HEK293A WT cells were transfected with siRF or siRNAs for AP3S1 subunit (#1 and #2) to deplete the AP-3 complex. Immunofluorescence was performed to follow ATG9A localization using anti-ATG9A, anti-GM130 antibodies and DAPI for nuclear staining. Scale bar: 10 µm. (D) Quantification of ATG9A on the Golgi. $n = 3$ independent experiments, * $p < 0.05$, ** $p < 0.01$. (E) Western Blot from HEK293A cells transfected with siRNAs for AP3S1 subunit. (F) siRF and siAP3S1#1 cells were treated with LLOMe 1 mM for 15 minutes. Immunofluorescence was performed using anti-ARFIP2 and anti-LAMP1 antibodies. Scale bar: 10 µm. (G) Quantification from (F), $n = 3$. (H) CTRL and CrARFIP2KO cells were treated with LLOMe 1 mM for 15 minutes followed by immunofluorescence using anti-AP3D1 and anti-LAMP1 antibodies. Scale bar: 10 µm. (I) Quantification from (H), $n = 5$.

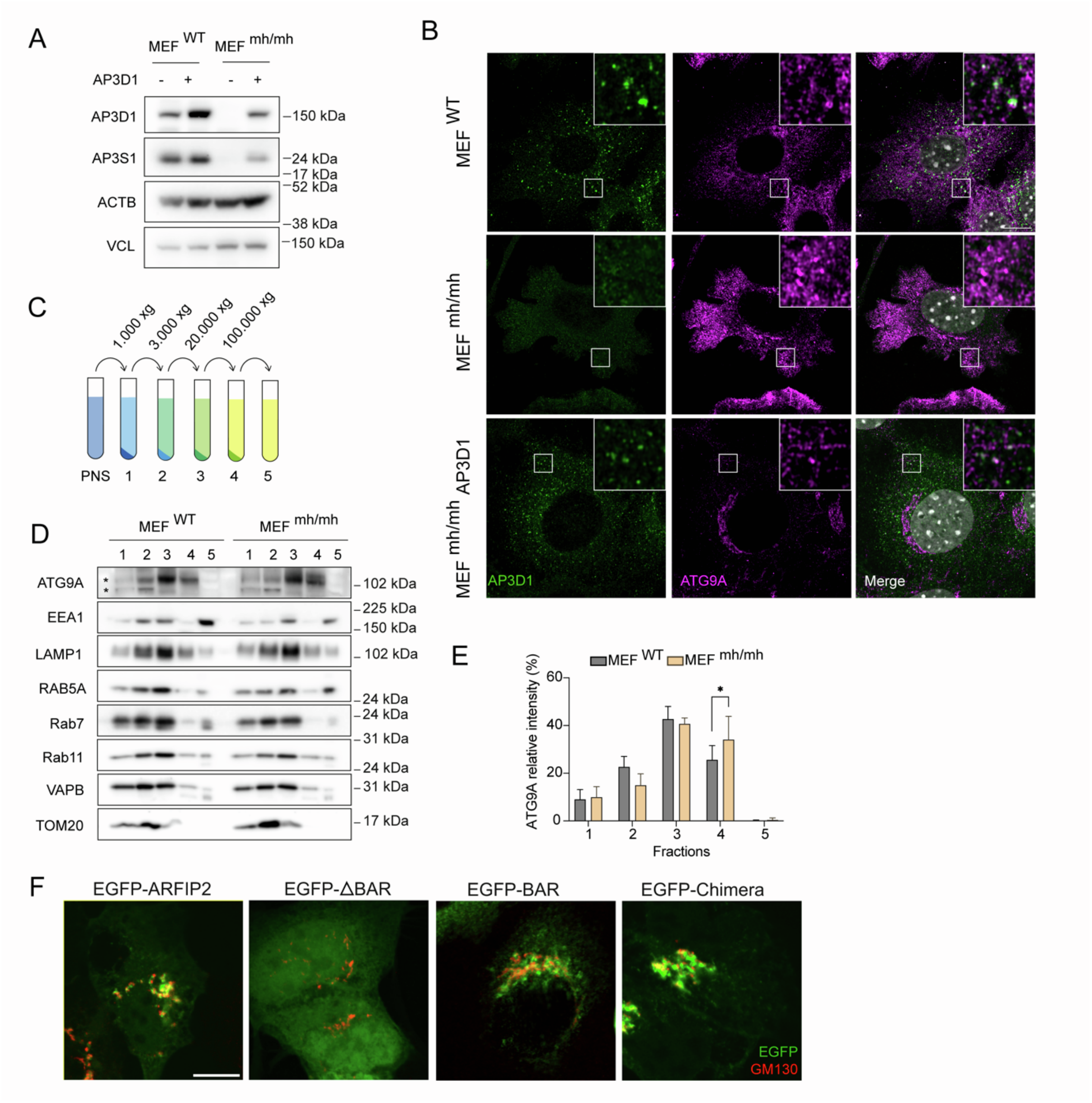

Figure S4. AP-3 regulates ATG9A trafficking and interacts with ARFIP2 N-terminal domain, related to Figure 4. (A) MEF^{WT}, MEF$^{mh/mh}$ and MEF$^{mh/mh}$ stably expressing AP3D1 subunit were subjected to Western Blot. (B) Immunofluorescence using anti-ATG9A and anti-AP3D1 antibodies and DAPI for nuclear staining in the indicated cell lines. (C) Schematic representing the differential centrifugation protocol used for membrane fractionation. (D) Western Blot analysis of organelle markers and ATG9A in MEF^{WT} and MEF$^{mh/mh}$ collected from the pellets. Asterisks (*) indicate the different glycosylated forms of ATG9A. (E) Quantification of the ATG9A proportion in the different fractions. n = 3 independent experiments, * $p < 0.05$. (F) CrARFIP2KO cells transiently expressing EGFP-ARFIP2, EGFP-ΔBAR (1-108 aa), EGFP-BAR (109-341 aa) and EGFP-ARFIP Chimera were processed for immunofluorescence using GM130 antibody to label the Golgi compartment. Scale bar: 10 µm.

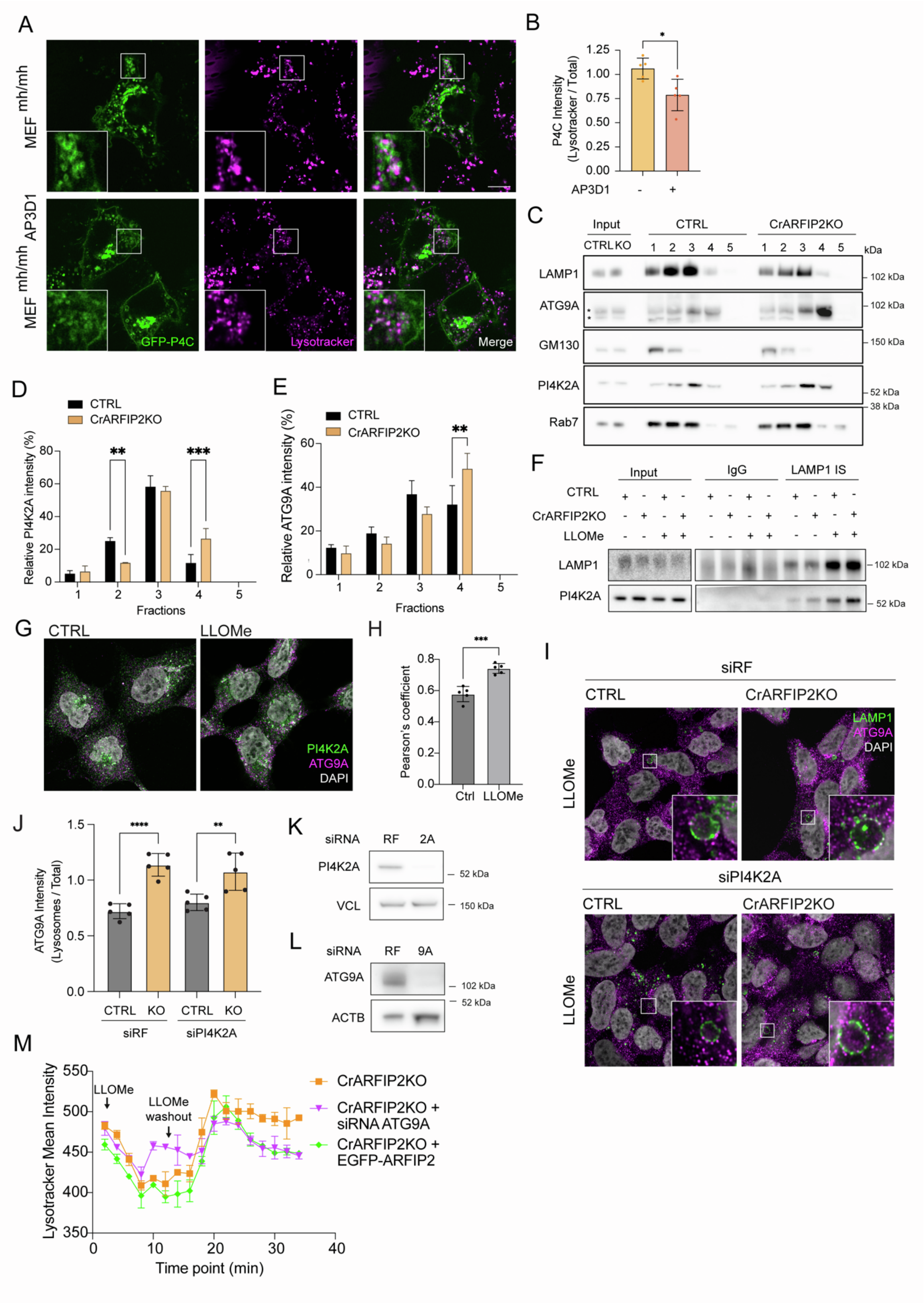

Figure S5. ATG9A and ARFIP2 control PI4K2A lysosomal trafficking, related to Figure 5. (A) MEF$^{mh/mh}$ and MEF$^{mh/mh}$ stably expressing AP3D1 subunit were transfected with GFP-

P4C for 24h. Live imaging was performed after incubation with Lysotracker for 1 h. Scale bar: 10 μm. (B) quantification of (A). $n = 5$, * $p < 0.05$. (C) Western Blot from CTRL and CrARFIP2KO cells after membrane fractionation using the indicated antibodies. (D) Quantification of the PI4K2A and (E) ATG9A proportion in the different fractions. $n = 3$ independent experiments, ** $p < 0.01$, *** $p < 0.001$. (F) Lysosome purification was performed using a LAMP1 antibody in CTRL and CrARFIP2KO cells treated with LLOMe for 15 minutes. Western Blot analysis performed using the indicated antibodies (G) HEK293A cells were treated with LLOMe for 15 minutes followed by immunofluorescence using anti-ATG9A and anti-PI4K2A antibodies. Scale bar: 10 mm. (H) Quantification of the Pearson correlation between ATG9A and PI4K2A from (H). $n = 5$ fields (100 cells), *** $p < 0.001$. (I) CTRL and CrARFIP2KO cells were transfected with siRF or siPI4K2A and treated with LLOMe for 15 minutes followed by immunofluorescence using anti-ATG9A and anti-LAMP1 antibodies. Scale bar: 10 μm. (J) Quantification of (I). $n = 5$, ** $p < 0.001$, **** $p < 0.0001$. (K-L) Western Blot showing depletion of PI4K2A (K) and ATG9A (L) upon siRNA treatment. (M) CrARFIP2KO, CrARFIP2KO treated with ATG9A siRNA for 72h and CrARFIP2KO cells transfected with EGFP-ARFIP2 were loaded with 25 nM Lysotracker DND-99 for 45 minutes. Cells were then treated with 1 mM LLOMe for 15 minutes, followed by wash out and the fluorescence recovery was measured. $n = 1$ independent experiment as average of 2 replicates and 6 fields per replicate.

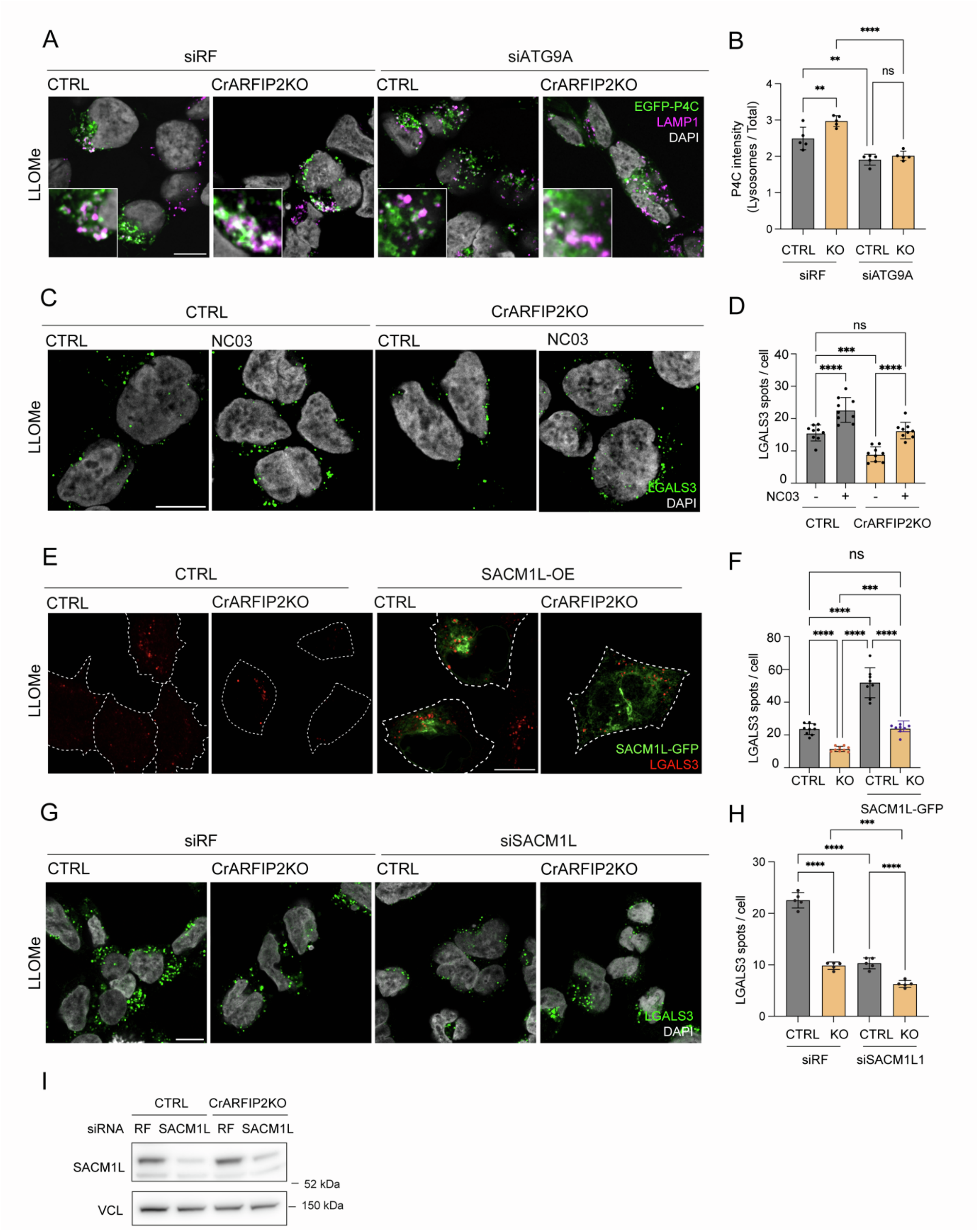

Figure S6. Enhanced lysosomal repair in CrARFIP2KO cells correlates with elevated PI4P production on lysosomes, related to Figure 6. (A) siRF or ATG9A siRNA was transfected into CTRL or CrARFIP2KO cells. After 48h, cells were transfected with GFP-P4C for 24h followed by treatment with LLOMe 1mM for 15 min. Immunofluorescence was performed using anti-LAMP1 antibody. Scale bar: 10 μm. (B) Quantification of (A), $n = 5$,

** $p < 0.001$, **** $p < 0.0001$. (C) CTRL or CrARFIP2KO cells were treated or not with 25 µM PI4K2A inhibitor NC03 for 1h and subsequently treated with 1 mM LLOMe for 15 minutes, with or without NC03. LGALS3 spots were detected by immunofluorescence. Scale bar: 10 µm. (D) Quantification of (C). $n = 3$ independent experiments, *** $p < 0.001$, **** $p < 0.0001$. (E) CTRL and CrARFIP2KO cells were transfected with SACM1L-GFP and subsequently treated with 1 mM LLOMe for 15 minutes. Immunofluorescence was performed using anti-LGALS3 antibody. Scale bar: 10 µm. (F) Quantification of (E). $n = 3$ independent experiments, *** $p < 0.001$, **** $p < 0.0001$. (G) CTRL and CrARFIP2KO cells were transfected with siRF or siSACM1L for 72h and treated with LLOMe for 15 minutes. Immunofluorescence was performed using anti-LGALS3 antibody. Scale bar: 10 µm. (H) Quantification of (G), $n = 5$, *** $p < 0.001$, **** $p < 0.0001$. (I) Western Blot analysis of cell lines from (G) using the indicated antibodies.

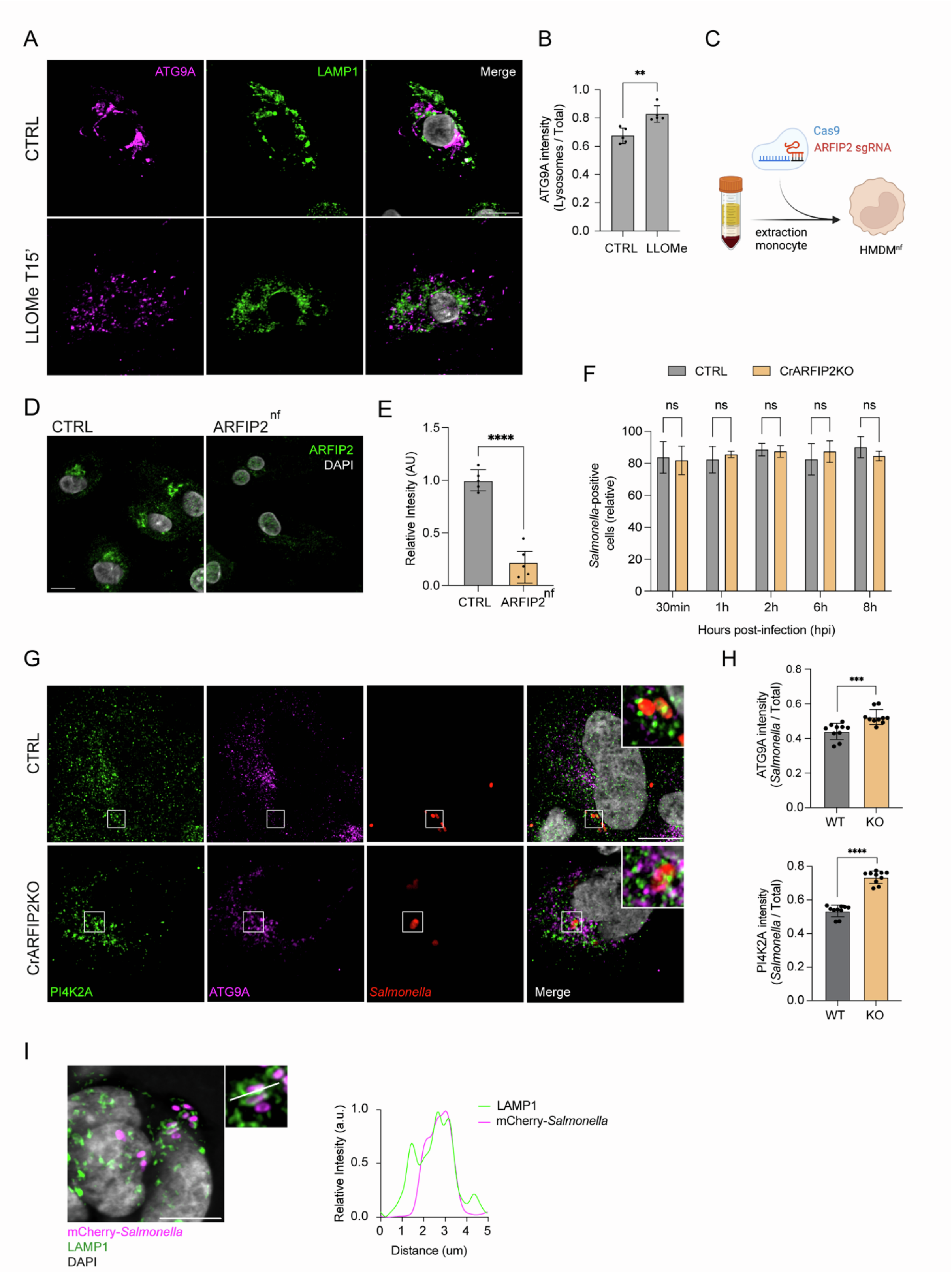

Figure S7. ARFIP2 regulates bacterial infection through lysosomal repair, related to Figure 7. (A) HMDM cells were treated or not with 1 mM LLOME for 15 minutes followed by immunofluorescence using anti-ATG9A and anti-LAMP1 antibodies and DAPI for nuclear

staining. Scale bar: 10 µm. (B) Quantification of ATG9A overlap with the lysosomal compartment is shown. n = 5 independent experiments, ** $p < 0.01$ (C) Schematic of the protocol used to generate HMDM cells nucleofected with CRISPR guides. (D) Representative images of CTRL and ARFIP2^{nf} HMDM cells. Immunofluorescence was performed using anti-ARFIP2 antibody. Scale bar: 10 µm. (E) Quantification of ARFIP2 fluorescence in CTRL and ARFIP2^{nf} HMDM cells. *n* = 5 fields, **** $p < 0.0001$. (F) Quantification of cells infected with *Salmonella* in CTRL and CrARFIP2KO cells at the indicated time points post-invasion. *n* = 3 independent experiments. (G) CTRL and CrARFIP2KO cells were infected with mCherry-*Salmonella* for 10 minutes. After 30 minutes, immunofluorescence was performed to detect ATG9A and PI4K2A recruitment to the pathogen. DAPI was used for nuclear staining. Scale bar: 10 µm. (H) Quantification of ATG9A or PI4K2A intensity on *Salmonella* bacilli. *n* = 5 independent experiments, *** $p < 0.001$, **** $p < 0.0001$. (I) Immunofluorescence analysis of *Salmonella* and LAMP1. (top) A line plot was used to represent bacteria engulfed in LAMP1-positive compartment after 30 min post-infection (right). Scale bar: 10 µm.
